# Assessment of Variations in Round Green Tea Volatile Metabolites During Manufacturing and Effect of Second-Drying Temperature *via* Nontargeted Metabolomic Analysis

**DOI:** 10.3389/fnut.2022.877132

**Published:** 2022-04-14

**Authors:** Huajie Wang, Yaya Yu, Wen Ouyang, Yongwen Jiang, Jinjin Wang, Jinjie Hua, Haibo Yuan

**Affiliations:** ^1^Key Laboratory of Tea Biology and Resources Utilization, Ministry of Agriculture, Tea Research Institute, Chinese Academy of Agricultural Sciences, Hangzhou, China; ^2^Department of Tea Science, College of Agriculture and Biotechnology, Zhejiang University, Hangzhou, China; ^3^State Key Laboratory of Tea Plant Biology and Utilization, College of Tea and Food Science and Technology, Anhui Agricultural University, Hefei, China

**Keywords:** round green tea, volatile metabolites, second-drying temperature, fatty acid-derived volatiles, glycoside-derived volatiles, nontargeted metabolomic analysis

## Abstract

Round green tea (RGT) is widely distributed and presents a high yield in China. The quality of RGT can be determined by its aroma; however, the transformation and formation of volatile metabolites during RGT processing remain unclear. In this study, 173 volatile compounds (nine categories) were identified totally from RGT *via* gas chromatography-mass spectrometry with infrared-assisted headspace-solid phase microextraction. These substances exhibited different changing trends during various procedures, with the most intense transformation occurring during fixation, followed by pan-frying and second drying; moreover, 51 substances were screened, mainly containing fatty acid-derived volatiles (i.e., (E)-2-hexen-1-ol, Hexanal, pentanal, hexanal) and glycoside-derived volatiles (i.e., linalool, geraniol, benzyl alcohol, benzaldehyde), and their evolution during processing was clarified. Furthermore, the effect of the second-drying temperature on volatile compound metabolism was clarified, and 90°C was the best temperature for RGT aroma. This research lays a foundation for in-depth quality control and the aroma formation mechanism of RGT.

## Introduction

Green tea has gained immense interest among consumers worldwide due to its green leaves and soup color, high fragrance, brisk and mellow taste, and various physiological activities such as anticancer, antioxidant, antiradiation, and antibacterial properties (1–4). Green tea can be classified into flat-shape, curled-shape, round (bead)-shape, needle (bud)-shape, and strip-shape, based on its appearance type (5). Round green tea (RGT), such as Yongxi Huoqing green tea in Anhui province, Fenghua curled tea and Pingshui Rizhu tea in Zhejiang Province, and Emerald tea in Guizhou Province, are widely consumed in China owing to their unique quality characteristics including tight, heavy, and sturdy round shape, green glossy color, high fragrance, and brisk and mellow taste (6, 7). Moreover, RGT accounts for a large proportion of green tea exported from China and is consumed by people worldwide.

Aroma is a key factor in evaluating the quality of green tea and affects its market value. The volatile compounds in green tea mainly include alcohols, aldehydes, ketones, esters, aromatic hydrocarbons, and terpenes (8). In tea, the volatile compounds arise from two sources—fresh tea leaves and substances formed during processing. These substances can be classified into four categories from different precursors: amino acid-derived volatiles, wherein amino acids undergo a Strecker reaction under the action of heat, and Maillard reaction with sugars to produce aldehydes, ketones, furans, pyrazines, and high-boiling volatile compounds (9, 10); glycoside-derived volatiles and glycoside-derived Non-volatiles, which release bound volatile compounds such as linalool and geraniol under the action of glycosidase or heat (11); lipid-derived volatiles, wherein lipids are first degraded to produce linolenic acid and other fatty acids, followed by production of lower-pointing alcohols and aldehydes (12); and carotenoid-derived volatiles, wherein carotenoids are precursors of volatile compounds such as ionone, 2,6,10,10-tetramethyl-1-oxaspiro[4.5]dec-6-en-8-one, and β-tanshinone (13).

The combination of processing technologies directly affects the formation pathways of volatile compounds and the type and quality of the green tea aroma (14). Moreover, tea leaves with distinct shapes made using different processing techniques have different cell breakage rates and material leaching rates, resulting in various aroma types, for example, flat and striped teas often exhibit floral and chestnut aromas, and needle-shaped teas often reveal delicate aromas (15, 16). Previous studies have reported the aroma of flat striped, needle-shaped, curly-shaped green teas (7, 17, 18); however, little research has been conducted on the aroma of RGT. The processing technology of RGT includes spreading, fixation, rolling, pan-frying, roll-roasting, and second-hot air drying. Among these, spreading promotes the loss of low-boiling compounds, such as (z)-3-hexen-1-ol and (E)-2-hexenal (19). Fixation may increase the formation of high boiling point substances, such as 3-methyl-butanal, linalool, and trans-β-ionone, under high temperature (8). Pan frying, the key process for shape formation and internal quality of RGT, is generally performed twice or thrice, with an initial increase in temperature and time, followed by a gradual decrease. Repeated pan frying helps to form round, tight, and heavy shape, and can trigger the biochemical reaction in the leaves under prolonged heat, which is conducive to the transformation and retention of quality components and lays the foundation for the formation of excellent quality. Roll-roasting and second-hot air drying promotes further emission of grass-flavored substances, formation of high-boiling substances, and the Maillard reaction of amino acids and soluble sugars to form heterocyclic substances such as furans and pyrazines at high temperatures (20).

The current research on RGT only focuses on the effect of processing technology on the sensory evaluation results and the total amount of physical and chemical components (21, 22). Non-targeted metabolomics is a novel analytical tool that allows the simultaneous determination of numerous substance components and comprehensive analysis of metabolites changes. It has been widely used in the analysis of Non-volatile and volatile metabolites of tea in recent years (23–25). However, few studies have reported the formation of aroma quality of RGT and volatile metabolite variations during manufacturing; moreover, the effect of RGT processing technology on the metabolism of volatile compounds remains to be elucidated. Therefore, this study uses the “Jiu Keng” fresh tea leaves as the raw material to process RGT and to obtain samples of the whole process (spreading, fixation, rolling, pan-frying, roll-roasting, and second drying). In addition, three second-drying temperatures (70°C, 90°C, and 110°C) were set to make RGT. Volatile compounds of these tea samples were detected using infrared-assisted-solid-phase microextraction (IRAE-HS-HPME) and gas chromatography-mass spectrometry (GC-MS) platforms. Multiple statistical analysis methods were used to elucidate the dynamic evolution of volatile compounds and the key metabolic pathways of volatiles biosynthesis during the processing of RGT, and the influence of different second-drying temperatures on volatile metabolites conversion. This study can provide a theoretical reference and objective basis for the mechanism underlying the formation of the aroma of high-quality RGT.

## Materials and Methods

### Experimental Materials

Fresh tea leaves (FTL) of JiuKeng (*Camellia sinensis L*.) were collected from the Zhejiang Kaihua tea production area (29°10′ N, 118°10′ E; at an elevation of 700 m a.s.l.) in April 2020. The moisture content of the FTL was ~78%. Each shoot comprised two leaves and one bud.

All the chromatography-grade volatile standards were purchased from Shanghai Yuanye Biological Technology Company (Shanghai, China). The purity of these chemical standards was ≥98%.

### Green Tea Processing

(1) FTL (25 kg) were spread 2–3 cm thickness on each wooden net plates (1 m × 1 m) for 12 h at the temperature 19–22°C with a relative air humidity of 60% environment until the moisture reached 70.0%.
(2) Fixation was performed using a roller fixation machine (80-cm type, electromagnetic roller fixation machine, Ningbo Yaojiangyuan Machinery Co., Ltd., Ningbo, China), the temperature of rolling was 300°C, the speed of rolling was 22 rpm, the fixation time was 120 s, and 120 kg of tea leaves were treated per hour. The fixated tea leaves (FTS) spreading and resurgence was conducted at 0.5 h on the wooden net plates.
(3) The cooled fixated tea leaves (FLS) were subjected to rolling for 30 min in a rolling machine (6CR-50 type, Zhejiang Shangyang Machinery Co., Ltd., Quzhou, China) using the following sequences: no-pressure rolling (5 min), light-pressure rolling (10 min), intermediate-pressure rolling (10 min), and no-pressure rolling (5 min).
(4) The rolled leaves (RTL) were pan-fired thrice in a double-pan roasting machine (6CCGQ-50 type, Zhejiang Shangyang Machinery Co., Ltd., Quzhou, China). First, RTL was treated for 20 min under a pan temperature of 210°C with a fried speed of 38 r/min until the leaves achieved a moisture content of 27.5%, as the pan-fried 1 leaves (PF1); thereafter, PF1 was treated for 40 min at a pan temperature of 180°C with a fried speed of 32 rpm until the leaves reached a moisture content of 15.0%, as the pan-fried 2 leaves (PF2); and PF2 was treated for 25 min at a pan temperature of 200°C with a fried speed of 32 rpm until the leaves had a moisture content of 10.0% (pan-fried 3 leaves (PF3).
(5) The pan-fried three leaves (PF3) were depilated for 15 min in a depilating machine (100-type, Zhejiang Shangyang Machinery Co., Ltd., Quzhou, China).
(6) The depilated leaves (DTL) were dried at 100°C for 15 min in a six-edge roasting machine (6CCP-60 type, Zhejiang Shangyang Machinery Co., Ltd., Quzhou, China) until 7.50% of leaf water content was achieved; thereafter, the leaves were spread out for a further 30 min, as the rotary pot first-dried leaves (RPL).
(7) Eventually, the samples were independently dried at 70°C, 90°C, and 110°C for 30 min until complete dryness was achieved in a box hot air-drying machine (Zhejiang Shangyang Machinery Co., Ltd., Quzhou, China); these dried at 70°C, 90°C, 110°C in box hot-air as the second-dried leaves (BHL70, BHL90, BHL110).

**Figure 1:**
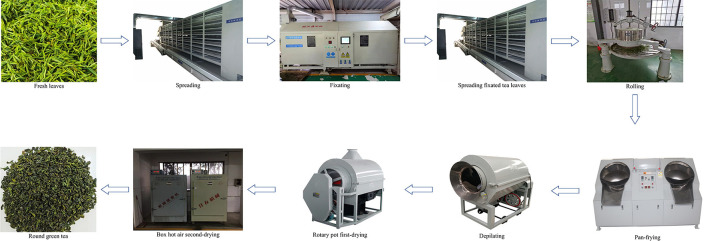
Flow chart of round green tea processing.

The overall production process of RGT is illustrated in Figure 1.

### Analysis of the Volatile Compounds Present in the RGT Samples

#### Sample Pretreatment

The green tea samples (0.5 g) were accurately weighed (0.001 g), and 20 mg/L ethyl caprate (10 μL) were added to a 20 mL headspace vial along with boiled distilled water (1 mL). The headspace vial cap was immediately tightened, and the protective cap was impaled using a DVB/CAR/PDMS fiber (2cm) head with a manual handle (SPME, Supelco, Commonwealth of Pennsylvania, USA). Thereafter, the vial was heated over a 100 W infrared (IR) device (Qiyi Lighting Company, Zhejiang, China) for 15 min, and the fiber head was inserted into the GC-MS inlet and desorbed at 250°C for 5 min. Each sample was subjected thrice to IR-assisted headspace-solid-phase microextraction (8).

#### GC-MS Analysis

The gas chromatograph (7890B−7000C, Agilent Technologies, Palo Alto, CA, USA) equipped with a HP-5 ms ultra-inert capillary column (30 m × 0.25 mm × 0.25 μm) was used in the splitless injection mode with a high-purity helium (99.999%) flow at 1.0 mL/min for GC-MS analysis. The GC injector temperature was maintained at 250°C, and the temperature program of following column was employed: initial temperature, 50°C (held for 5 min), increased to 150°C at a rate of 4°C/min (held for 2 min), and increased to 270°C at a rate of 10°C/min (held for 6 min) (26, 27).

#### Qualitative and Quantitative Analyses

For qualitative analysis, the volatile metabolites with a similarity of >80% to the NIST11 standard library (22) were screened using the Agilent Mass Hunter unknown analysis program. Kovàts retention indices for each compound were obtained by calculating the linear formula of n-alkanes (C7-C40) and by comparing with theoretical retention indices referred to the literature value with the same or equivalent chromatographic column on the NIST Chemistry WebBook (http://webbook.nist.gov/chemistry/), based on the difference of RI within 30 (8). Moreover, some key aroma substances, such as 1-hexanol, (E)-4-heptenal, octanal, 1-decanol, linalool, naphthalene, decanal, geraniol, indole, α-ionone, cedrol, trans-β-ionone, hexadecenoic acid ethyl ester, and linoleic acid ethyl ester were identified further with authentic standards. For quantitative analysis, the mass concentrations of the volatile metabolites were calculated regarding the internal standard method using equation:


(1) Ci = (Cis × Ai)/Ais

*C*_*i*_ represents the concentration of any volatile metabolites (μg/L), *C*_*is*_ is the concentration of the internal standard (μg/L) (ethyl caprate, 20 mg/L), *A*_*i*_ is the chromatographic peak area of any metabolites, and *A*_*is*_ represents the chromatographic peak area of the internal standard (27).

##### OAV

The OAV is the ratio of the concentration of volatile compounds in a sample to the odor threshold (28), calculated according to equation:


(2) OAV = c/OT

c is the concentration of the metabolites in the green tea sample (μg/L), and OT represents the odor threshold of the corresponding components in water (μg/L).

### Statistical Analysis

The tests were repeated in triplicate, and the results of each test were expressed as the average of three replicates. The analyze the principal components affecting the volatile metabolites according to the different green tea processing using SIMCA P13 software (Umetrics, Umea, Sweden). MEV 9.0 software (https://mev-tm4-org.caas.cn) was used to generate heatmaps of key differential metabolites. SAS software (version 9.4; SAS Institute Inc., Cary, USA) was used to analyze the significant differences in the concentration of volatile compounds according to different treatments.

## Results

### Analysis of Volatile Compounds During the Processing of Green Tea

#### Overview of the Profile of Volatile Compounds in RGT

In total, 173 volatile compounds were identified (Supplementary Table S1) in this study, including 32 alcohols, 14 aldehydes, 17 ketones, 31 esters, 16 terpenes, 15 aromatic hydrocarbons, 28 alkanes, 3 furans, and 17 other types.

**Figure 2:**
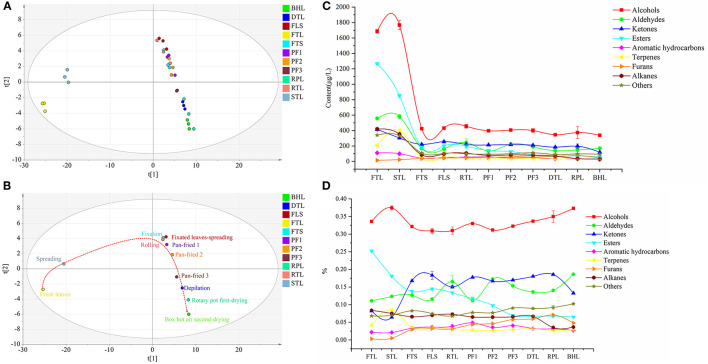
**(A)** Principal Component Analysis (PCA) score scatter plot composed of all volatile compounds in the round green tea process. **(B)** The dynamic metabolic trajectory plot of volatile compounds, drawn by scatting of the Principal Component Analysis (PCA) scores t[1] vs. t[2]. **(C)** Variation trend chart of different types of volatile compounds content in different processes of round green tea. **(D)** Variation of the proportion of different types of volatile compounds in different process of round green tea.

Moreover, a Non-supervised metrology PCA tool was used to conduct a comprehensive analysis of these 173 volatile compounds, as depicted in the score plot (Figure 2A). All tea samples revealed marked separation and clustering. FTL and STL are on the left side of the score plot, whereas the samples of FTS and subsequent samples are on the right. The trajectory plot (Figure 2B) clearly depicts stepwise alterations and distinct differences in volatile compounds from fresh leaves to finished leaves. Furthermore, a great change was observed in volatile compounds after fixation, and a further shift occurred in the pan-fried and box hot air drying; however, minor variations were observed during rolling, depilation, and rotary pot first-drying.

### Dynamic Changes of Volatile Categories During RGT Processing

Nine categories of volatile compounds underwent remarkable changes during RGT processing. Changes in the content of different categories of volatile compounds are illustrated in Figure 2C; the contents of alcohols and aldehydes slightly increased in STL and markedly decreased in FTS; that of aldehydes significantly increased in RTL and PF2 and then decreased (*p* < 0.05); and the ketones markedly decreased in STL, FTS, and BHL, and increased in FLS and RPL. The ester content decreased throughout the process, and the sharpest decline was observed after fixation; terpenes increased in STL, significantly decreased in FTS, and slightly fluctuated in FLS (*p* < 0.05); and aromatic hydrocarbons revealed an alternate decreasing and increasing trend in FTS. The content of furans initially increased before the third pan-fried process and then decreased in DTL and BHL. The contents of alkanes and others (such as benzoic acid, hydrazide, benzyl nitrile, and 2-hexyl-5-pentylpyrrolidine) showed downward trends, particularly in fixation. The total volatile compound content revealed following trend: FTL > STL > RTL > FLS > FTS > PF2 > PF3 > PF1 > RPL > DTL > BHL (Supplementary Table S1). From the proportion of categories (Figure 2D), alcohols accounted for the highest proportion in the whole process (30.9–37.5%); moreover, the proportion of aldehydes increased significantly in RTL, PF2, and BHL (*p* < 0.05), whereas that of esters decreased in the process (*p* < 0.05). The proportion of ketones increased significantly in the FTS and decreased in the BHL (*p* < 0.05). The terpenes increased in the STL and then decreased. It is speculated that the activity of glycoside hydrolase increased during spreading, thereby promoting the release of terpene volatile compounds. Furans and aromatic hydrocarbons occupied a small proportion in the whole process (0.9–4.8%).

### Differential Compound Screening and Evolution Analysis During RGT Processing

**Figure 3:**
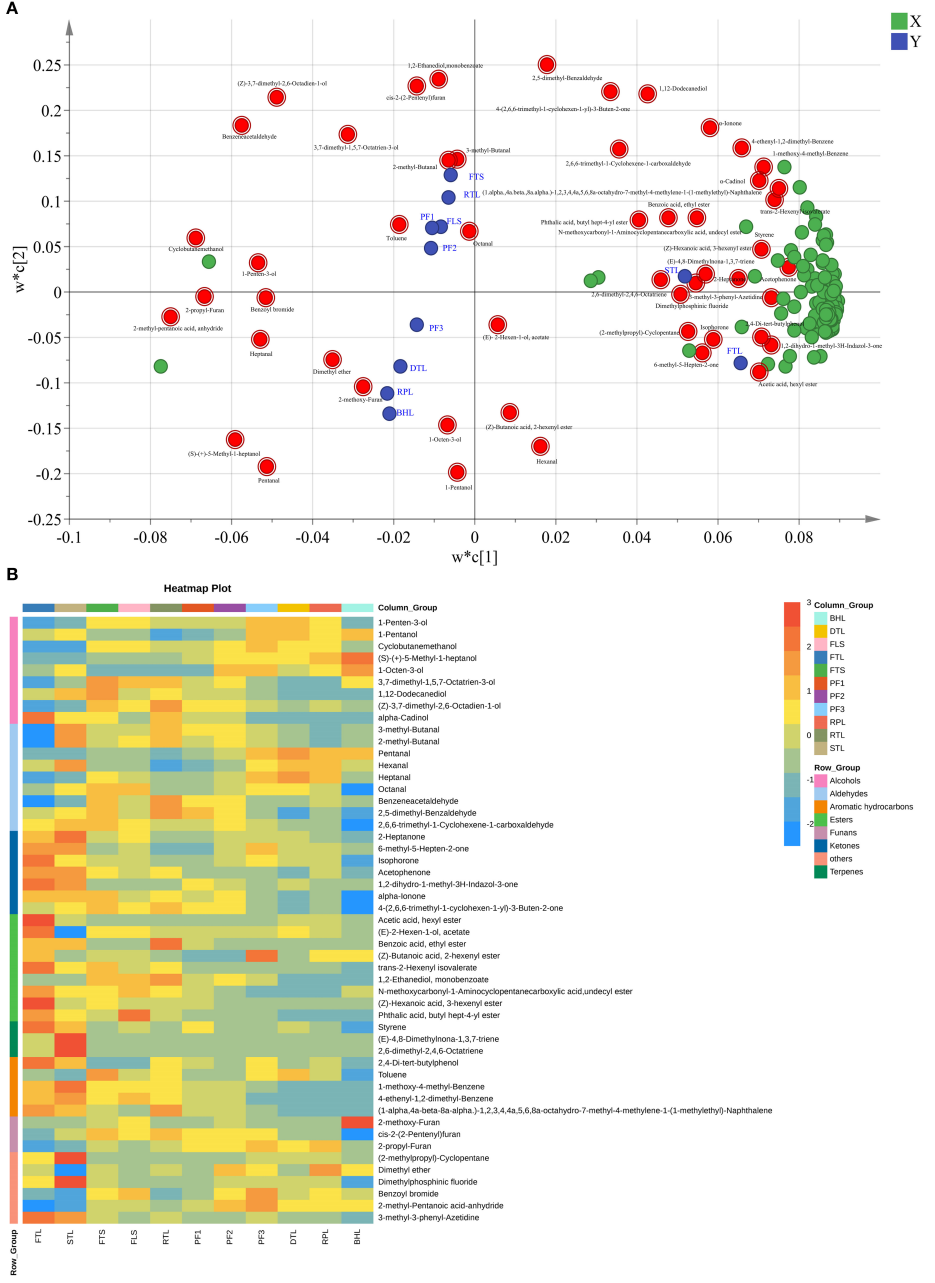
**(A)** Loading diagram of Partial Least Squares Discrimination Analysis (PLS-DA) model. **(B)** Heat map of 51 difference volatile compounds.

Analysis of the volatile compounds indicate that volatile compounds undergo drastic changes during processing. To clearly distinguish the important compounds in the process and to determine the law of their evolution, 51 substances were screened *via* variable projection importance (VIP) in the PLS-DA analysis (VIP > 1) and one-way analysis of variance (*p* < 0.05), as depicted in the loading diagram of PLS-DA (Figure 3A). Moreover, 51 substances mainly include alcohols, aldehydes, esters, ketones, aromatics hydrocarbons, terpenes, and furans, such as (4E)-4-hexen-1-ol, acetate, hexanal, heptanal, octanal, benzene acetaldehyde, 3-methyl-butanal, 2-methyl-butanal, benzene acetaldehyde, 2,5-dimethyl-benzaldehyde, 2-heptanone, α-cadinol, 3,7-dimethyl-1,5,7-octatrien-3-ol, 1-penten-3-ol, 1-pentanol, (E)-2-hexen-1-ol, acetate, styrene, (Z)-hexanoic acid, 3-hexenyl ester, α-ionone, and isophorone (Figure 3B).

### Evolution of Key Different Compounds and Their Related Compounds During Processing

From the 51 compounds obtained in section Differential Compound Screening and Evolution Analysis During RGT Processing, the metabolic transformation rules of 34 key volatile compounds with definite aroma characteristics (9, 13) were revealed. Combined with the transformation and metabolism-related substances, this study clarified the evolutionary law of total 48 volatile compounds in the process of RGT, which contained 21 fatty acid-derived volatile substances, 18 glycoside-derived volatile substances, 7 amino acid-derived volatile substances, and 3 carotenoid-derived volatiles (Supplementary Figure S1).

#### Fatty Acid-Derived Volatiles

**Figure 4:**
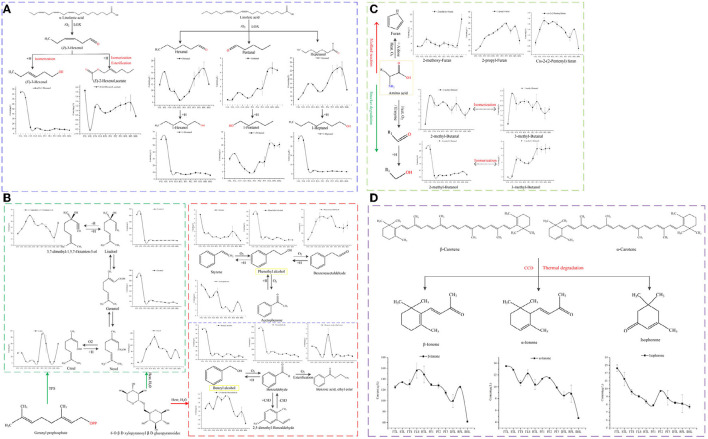
Evolution of compounds from different sources: **(A)** Fatty derived volatiles, **(B)** Glycoside-derived volatiles, **(C)** Amino acid-derived volatiles, and **(D)** Carotenoid-derived volatiles.

Lipids are important precursors for the formation of volatile compounds in green tea. The degradation products of unsaturated fatty acids or saturated fatty acids, such as α-linolenic acid and linoleic acid, can form C6-aldehydes under the action of heat or lipoxidase (LOX), and then further undergo redox reaction, isomerization, and may form esters, as well as chemical reactions to generate corresponding ketones, and alcohols (8). Figure 4A illustrates such compound changes during processing of RGT: under the action of lipoxidase, the hexanal, pentanal and heptanal derived from linoleic acid showed an upward trend, whereas (E)-2-hexen-1-ol, acetate decreased sharply. Hexanal, 1-hexanol, 1-pentanol, 1-heptanol and (E)-3-hexen-1-ol derived from linoleic acid and α-linolenic acid decreased significantly after fixating, whereas (E)-2-hexen-1-ol, acetate showed increased trend. During the spreading process, under the action of hydrolases, oxidases, and isomerases, hydrolysis of esters is increased, and the degradation of lipids results in the formation of corresponding alcohols and aldehydes, under the heat of fixation, the oxidation and esterification were promoted, these results are consistent with the existing findings (19, 29). High boiling point compounds, such as pentanal, heptanal, hexanal, and 2-heptanone, 6-methyl-5-hepten-2-one, and 1-penten-3-ol (Supplementary Figure S1) increased significantly during pan-fried process (*p* < 0.05), likely because the process of rolling promoted the fragmentation of tea tissue cells, resulting in a large amount of unsaturated fatty acids overflowing. Thus, these unsaturated fatty acids underwent strong thermal oxidation reactions under long-term high temperature treatment of pan-fried to form alcohols, aldehydes with high boiling points. Furthermore, heat fixation and pan frying increased the volatilization of low-boiling alcohols and induce the formation of corresponding aldehydes, ketones esters by oxidation, isomerization, esterification reactions.

#### Glycoside-Derived Volatiles

Glycoside-derived volatiles generally exhibit floral, sweet, and fruity aromas and form an important component of green tea aroma (11). During the process, monoterpene alcohols (linalool, and geraniol) or aromatic alcohols (benzyl alcohol and phenylethanol) can be liberated by glycosidic enzymes or heat reaction. In this study, the levels monoterpene alcohols, such as linalool, geraniol markedly decreased after fixation (Figure 4B), whereas the dehydrogenation compound (3,7-dimethyl-1,5,7-octatrien-3-ol), isomerisation compound (nerol) considered important components of the floral and fruity aroma of green tea (28), remarkably increased. The levels of methyl salicylate, alfa-copaene, cubenene, α-cadinol, etc., (Supplementary Figure S1) markedly decreased in FTS, whereas those of citral, as the oxide of nerol, which is the isomer of geraniol and linalool, were significantly increased in PF2 and BHL, particularly in PF2 (*p* < 0.05). This indicates that the isomerization of linalool, geraniol and the oxidation reaction of nerol may generate citral under long-term high-temperature treatment of PF2 (11). The level of aromatic compounds, such as benzyl alcohol, benzaldehyde, styrene, phenylethyl alcohol markedly decreased after fixation, while corresponding oxides (benzene acetaldehyde) was increased. Benzoic acid, ethyl ester was increased significantly after rolling process, which may relate to the increase of cell fragmentation rate under the action of external force and high leaching rate. The high temperature treatment of RPL promoted the oxidation and methylation reaction to form acetophenone, 2,5-dimethyl-benzaldehyde. Many glycoside-derived volatiles disappeared during the fixation process, which is consistent with the results of previous studies (11). It is speculated that in a high-temperature environment, some volatiles are lost and recombine with glycosides to form nonvolatile compounds; furthermore, they undergo oxidation, isomerization, and other reactions (8).

#### Amino Acid-Derived Volatiles

Amino acid-derived volatiles are generally derived from the Strecker reaction of amino acids and the Maillard reaction of amino acids with sugars (9). Acid-derived volatiles were identified in this study (Figure 4C). Among these, 2-methyl-butanal and 3-methyl-butanal increased significantly after spreading (*p* < 0.05), which is related to the reaction of leucine and isoleucine with glutamine under the action of transaminase and decarboxylase. After fixation, the levels of 3-methyl-butanal, 2-methyl-butanal, and 2-methyl-butanol were significantly reduced (*p* < 0.05), whereas those of 3-methyl-butanol significantly increased (*p* < 0.05). Moreover, at high temperatures, oxidation reaction was triggered, and strong reduction reaction and isomerization reaction also occurred (10); however, their contents in PF3 and BHL have rebounded, which is related to the high temperature and long-term treatment at this stage to promote the Strecker reaction of amino acids and oxidation and isomerization. 2-methoxy-furan, cis-2-(2-pentenyl) furan, and 2-propyl-furan are the Maillard reaction products; the levels of 2-methoxy-furan increased in FTS, PF2, BHL, particularly in BHL, which is consistent with the results of the existing research (9), and 2-propyl-furan exhibited an upward trend in most processes but decreased in DTL and BHL. Moreover, cis-2-(2-pentenyl) furan increased in STL, FTS, and RPL, whereas it decreased in BHL significantly (*p* < 0.05), which contradicts the existing research that high temperature and long-term drying is conducive to the Maillard reaction (30), and may be related to the further transformation of these two substances at high temperatures.

#### Carotenoid-Derived Volatiles

Carotenoids can generate ionone, 2,6,10,10-tetramethyl-1-oxaspiro [4.5] dec-6-en-8-one, and other floral and fruity substances under enzymatic oxidation and heat, and are important precursors of the formation of green tea aroma (9). The evolution of β-ionone, α-ionone and isophorone is depicted in Figure 4D; β-ionone, α-ionone increased during FLS and RPL, and the decrease was most obvious at BHL (29). Isophorone is another carotenoid-derived volatile (31), which exhibited decreased trend before PF2, but increased mostly in PF2 and further decreased. The rebound in PF1 and PF2 is related to the increase in cell disruption and the overflow of its precursor substances, which further promotes its synthesis under the action of high temperature (32).

### Effect of Box Hot Air Second-Drying Temperature on Volatile Compounds

In section Analysis of Volatile Compounds During the Processing of Green Tea discussion, it was found that the hot air drying process is crucial in the formation of aroma during RGT processing. Therefore, this study will further discuss the influence of different second-drying temperatures on the volatile compounds of RGT.

#### Volatile Profile of Green Tea Under Different Box Hot Air Second-Drying Temperature

**Figure 5:**
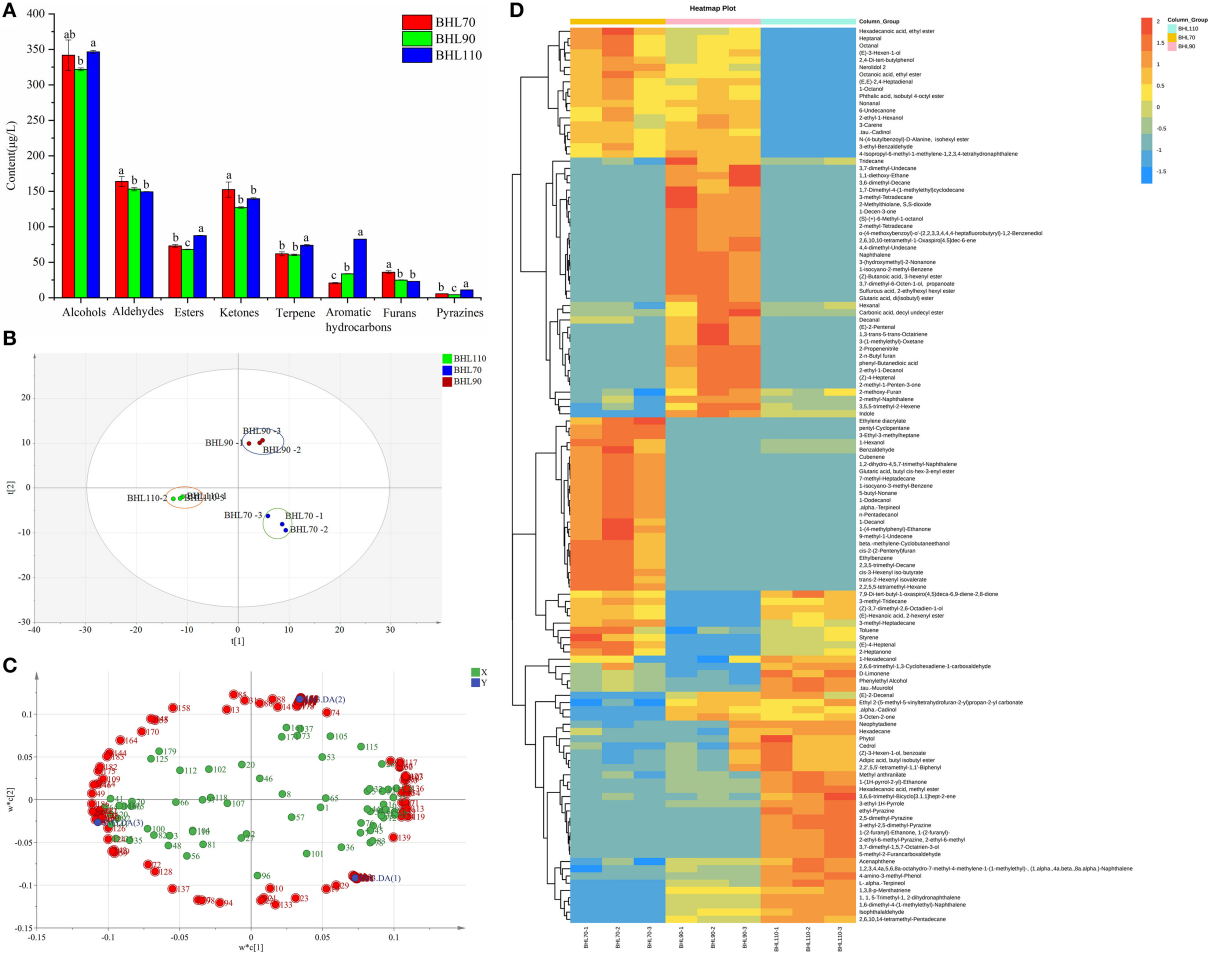
**(A)** Histogram of different types of volatile compounds of round green tea by different second-drying temperature. **(B)** Partial Least Squares Discrimination Analysis (PLS-DA) score. **(C)** Loading diagram for volatile compounds of round green tea by different second-drying temperature. **(D)** Heat map of 117 difference volatile compounds. Different letters indicate the significance of the difference of the same type substance at different temperatures through LSD test (*p* < 0.05).

In total, 247 volatile compounds were obtained in different second-drying temperature samples (Supplementary Table S2). The comparison of different temperature samples revealed that alcohols and furans were significantly higher in BHL70 (*p* < 0.05), whereas alkanes, terpenes, aromatic hydrocarbons, and pyrazines were higher in BHL110 than in other samples, and the contents of various compounds (such as alkanes, terpenes, aromatic hydrocarbons, alcohols, and furans) were moderate in BHL90 (Figure 5A). Substances with green aroma, such as (E)-3-hexen-1-ol, 1-hexanol, 1-decanol, hexanal, (E)-2-hexenal, and (E)-4-heptenal were higher in BHL70; naphthalene, 2-methoxy-furan, (E)-2-pentenal, 3-octen-2-one, and 2,6,10,10-tetramethyl-1-oxaspiro [4.5] dec-6-ene were higher in BHL90; whereas 3-ethyl-1H-pyrrole, 2-ethyl-6-methyl-pyrazine, 3-ethyl-2,5-dimethyl-pyrazine, and roast aroma were higher in BHL110 than BHL90 and BHL70 (Supplementary Table S2).

##### PLS-DA

To further clarify the effect of the second-drying temperature on volatile compounds and obtain key differential compounds, PLS-DA analysis (33) was conducted in this study based on 247 volatile compounds. The score map is illustrated in Figure 5B, and three groups of samples were distinguished. While detecting t [1], BHL70 and BHL90 were on the right side of the figure, and BHL110 was on the left side of the figure. The model parameters were R^2^X = 0.941, R^2^Y = 0.998, and Q^2^ = 0.988, indicating that the model is stable and reliable and has strong predictive ability.

To obtain the key volatile compounds that distinguish samples at different second-drying temperatures, variable importance of projection (VIP) and difference significance analysis were used to screen differential substances. In total, 117 substances (load diagram, Figure 5C), such as benzaldehyde, octanal, D-Limonene, naphthalene, hexanal, 3-ethyl-1H-Pyrrole, (E)-3-Hexen-1-ol, 2,5-dimethyl-pyrazine, and trans-β-ionone, were obtained. Supplementary Figure S2 illustrates the changes in 117 substances under different second-drying temperatures. The heatmap (Figure 5D) showed the changes of 117 differential compounds under different second-drying treatments clearly. Octanal, (E, E)-2,4-heptadienal, 1-Octanol, nonanal, and (E)-4-heptenal, with a grassy aroma, were higher in BHL70; 2-methoxy-furan, (E)-2-pentenal, 3-octen-2-one, and 2,6,10,10-tetramethyl-1-oxaspiro [4.5] dec-6-ene were higher in BHL90; and 3-ethyl-1H-pyrrole, 2-ethyl-6-methyl-pyrazine, and 3-ethyl-2,5-dimethyl-pyrazine were higher in BHL110. This result follows the previous studies reporting that significantly lower temperatures lead to greater retention of grass-flavored substances and insufficient formation of high-boiling aromatic substances (*p* < 0.05), whereas higher temperature can significantly promote the Maillard reaction of amino acids and sugars to produce too many pyrazines, which imparts a baking flavor to the tea (34).

##### OAV

OAV analysis is frequently applied to evaluate the contribution of volatile compounds in aroma (27, 35). Odor thresholds and characteristics of the 41 patients (Supplementary Table S3) consuming the 117 substances were obtained based on previous studies (8, 36–39). Of these, 21 substances (Table 1), with important contributions to aroma, were screened out according to the principle of OAV > 1. In particular, the OAV of trans-β-ionone is >9,000, and 1-octanol is >450, both of which are important components of aroma composition (8, 38). From the perspective of different second-drying temperature treatments, the OAVs of 1-hexanol, (E)-4-heptenal, octanal, 1-octanol, nerolidol 2, benzaldehyde with green, and flower aroma were higher in BHL70 than in the other treatments, whereas naphthalene, 2,6,10,10-tetramethyl-1-oxaspiro [4.5] dec-6-ene and decanal significantly contributed to BHL90 (*p* < 0.05). These compounds, such as 3,7-dimethyl-1,5,7-octatrien-3-ol, 2,5-dimethyl-pyrazine, ethyl-pyrazine, 2-ethyl-6-methyl-pyrazine, and 3-ethyl-2,5-dimethyl-pyrazine with roasted aroma are only present in BHL110; however, due to the high threshold of these substances, their OAV <1, but the relatively low OAV values of other substances highlight the baking flavor.

**Table 1:** Aroma characteristics and OAV values of 21 different volatile compounds (OAV > 1) in different second-drying temperature samples.

**Name**	**OT μg/L)^A^**	**Aroma characteristics^B^**	**BHL70**	**BHL90**	**BHL110**
1-Hexanol	0.7	Green, cut grass	10.14 ± 1.89a	2.37 ± 0.259b	4.13 ± 0.40b
2-Heptanone	1	pieplant, musty	2.15 ± 0.61a	0.00 ± 0.00c	1.34 ± 0.15b
(E)-4-Heptenal	0.02	Green	67.08 ± 15.33a	0.00 ± 0.00c	28.55 ± 2.95b
Heptanal	3	Heavy, planty green odor	5.94 ± 1.25a	3.98 ± 0.62b	0.71 ± 0.05c
Benzaldehyde	3	almond-like smell	2.27 ± 0.29a	1.04 ± 0.09b	1.24 ± 0.03b
Octanal	0.7	Green, fatty, citruse	5.88 ± 1.49a	3.96 ± 0.54b	0.00 ± 0.00c
1-Octanol	0.022	Green, citrus, fatty, coconut-like	533.65 ± 89.58a	450.99 ± 37.79b	0.00 ± 0.00c
Nonanal	1	candle-like, sweet orange-like, fatty and floral	29.97 ± 4.34a	29.62 ± 1.38a	0.00 ± 0.00b
Decanal	0.1	Aldehyde-like, candle-like, fatty and citrus-like aroma	18.96 ± 2.78b	27.89 ± 2.26a	16.44 ± 0.34b
1-Decanol	0.023	Orange, floral	25.13 ± 7.39a	0.00 ± 0.00b	0.00 ± 0.00b
Nerolidol 2	0.25	Floral, green, citrus, woody, waxy	11.08 ± 0.74a	6.98 ± 0.67b	0.00 ± 0.00c
tau-Cadinol	0.44	Tar, camphor, and greasy	5.42 ± 0.56a	5.49 ± 0.44a	0.00 ± 0.00b
Naphthalene	0.44	Pungent, dry, tarry odor	0.00 ± 0.00b	4.57 ± 0.09a	0.00 ± 0.00b
2,6,10,10-tetramethyl-1-Oxaspiro [4.5] dec-6-ene	0.2	Fruity, woody, slightly camphor-like	0.00 ± 0.00b	6.25 ± 0.11a	0.00 ± 0.00b
3,7-dimethyl-1,5,7-Octatrien-3-ol	110	Moldy	0.00 ± 0.00b	0.00 ± 0.00b	0.63 ± 0.03a
2,5-dimethyl-Pyrazine	1,750	Roasted	0.00 ± 0.00b	0.00 ± 0.00b	0.001 ± 0.00a
ethyl-Pyrazine	4,000	Nutty coffee, cocoa-like	0.00 ± 0.00b	0.00 ± 0.00b	0.00 ± 0.00a
5-methyl-2-Furancarboxaldehyde	500	Caramel, bready, coffee-like	0.00 ± 0.00b	0.00 ± 0.00b	0.01 ± 0.00a
2-ethyl-6-methyl-Pyrazine	40	Roasted	0.00 ± 0.00b	0.00 ± 0.00b	0.11 ± 0.00a
3-ethyl-2,5-dimethyl-Pyrazine	8.6	Roasted potato, cocoa-like, nutty	0.00 ± 0.00b	0.00 ± 0.00b	0.41 ± 0.01a
trans-beta-Ionone	0.007	Violet-like, floral, and raspberry-like	9,676.94 ± 1,134.50a	9,260.63 ± 769.57a	10,176.88 ± 592.00a

*Different letters (a, b, c) represent the significance of the difference between the same substance at different second drying temperatures through LSD test (p < 0.05)*.

*OTs: Odor thresholds in water. The values were calculated according to reported references*.

A *(8, 24, 25, 27, 31–34)*.

B *http://www.thegoodscentscompany.com/search3.php?qOdor=20126-765&submit.x=9&submit.y=9*.

## Discussion

Processing technology and second-drying temperature are key factors in the formation of RGT aroma (8, 39). In this study, the concentration of all the identified volatile compounds have undergone substantial changes during tea processing, particularly during the fixation process, followed by pan-frying and second-drying, which is consistent with previous reports (7, 8). Under the high-temperature fixation treatment, many low-boiling point alcohols, aldehydes, and esters are lost and transformed, and violent degradation, isomerization, redox, and other reactions occur, consequently forming high-boiling point compounds. During the pan-fried process, the tea leaves and pot wall are repeatedly rubbed. Therefore, the cell fragmentation rate increases, and the volatile compounds and their precursors are further released from the cells. Moreover, high temperature increases thermochemical reactions and promotes the formation of furans, aldehydes, and aromatic hydrocarbons with high-boiling point. During the box hot air second-drying, few low-boiling volatile compounds were further lost. This could be related to the large consumption of precursor compounds in fixation and pan-frying process. The levels of fatty acid-derived volatiles and glycoside-derived volatiles have undergone considerable changes during RGT processing, especially during the fixation process. Under high temperature condition during the fixation process, the temperature of tea leaves changes from 20 ~ 25°C to 70 ~ 80°C, the moisture content of tea leaves changes from 70 to 30% ~ 40%, the state of enzymes changes from active to inactive, and a series of enzymatic chemical reactions and thermochemical reactions take place in the tea leaves in 2 ~ 5 min. High temperature can promote the degradation of lipids to generate free fatty acids, which generate alcohols and aldehydes volatiles under the effect of heat-induced oxidation. The increase in glycoside-derived volatiles in fixation process may be due to the simultaneous enzymatic hydrolysis and thermochemical degradation of glycosides. This result is consistent with the previous reports that lipids and glycosides undergo drastic changes during fixation (11, 29, 40).

The second-drying temperature also had a great effect on the levels of volatile compounds. When the temperature was too low (70°C), the dry-heat reaction was insufficient and several green grass-flavor alcohols and aldehydes were retained (11). Therefore, the tea sample exhibited a faint scent. However, when the temperature was too high (110°C), the dry-heat reactions (Maillard reaction, Caramelization reaction, etc.) were too intense and several baking-flavor volatiles were generated (34). At the appropriate temperature (90°C), the content of each compound was moderate and the proportion of various types of volatile compounds was coordinated. In brief, the temperature at which biochemical reactions occur at a moderate rate to avoid overfired flavor is the best temperature to produce high-quality RGT.

## Conclusions

In this study, the evolution of volatile compounds during the processing of RGT was investigated using IR-HS-SPME technology combined with GC-MS, and the effects of different second-drying temperatures on the formation of volatile compounds were clarified. Fixation is considered the most drastic process of volatile metabolite conversion, followed by pan-fried and box hot air second-drying. Moreover, 51 key volatile substances were screened out, and fatty acid-derived volatiles and glycoside-derived volatiles changed most dramatically during processing. Substances with excellent aroma flavor, such as nonanal, decanal, tau-cadinol, naphthalene, and 2,6,10,10-tetramethyl-1-oxaspiro [4.5] dec-6-ene were significantly higher under BHL90 treatment (*p* < 0.05) and 90°C was the most suitable temperature for the second-drying processing of RGT. This study provides a theoretical basis and technical guidance for the processing of high-quality RGT and lays a foundation for in-depth exploration of the aroma formation mechanism during green tea processing. As only the precursors of part volatile metabolites are known, the number of classified volatile compounds discussed in this study is limited. In future research, we need to assess the volatile precursor substances, analyze the relationship between Non-volatile compounds and volatile compounds, and explore the mechanism of RGT aroma formation.

## Data Availability Statement

The original contributions presented in the study are included in the article/Supplementary Material, further inquiries can be directed to the corresponding authors.

## Author Contributions

HW: conceptualization, investigation, formal analysis, software, writing–original draft, and editing. YY: investigation, resources, and writing–review and editing. WOY and JW: investigation. YJ: data curation software. JH: project administration, writing–review, and editing. HY: funding acquisition and supervision. All authors contributed to the article and approved the submitted version.

## Funding

This work was supported by the Science and Technology Innovation Project of the Chinese Academy of Agricultural Sciences (CAAS-ASTIP-TRICAAS), the open fund of State Key Laboratory of Tea Plant Biology and Utilization (SKLTOF20210110), and the National Science Foundation of China (31972465).

## Conflict of Interest

The authors declare that the research was conducted in the absence of any commercial or financial relationships that could be construed as a potential conflict of interest.

## Publisher's Note

All claims expressed in this article are solely those of the authors and do not necessarily represent those of their affiliated organizations, or those of the publisher, the editors and the reviewers. Any product that may be evaluated in this article, or claim that may be made by its manufacturer, is not guaranteed or endorsed by the publisher.

## Abbreviations

FTL: Fresh tea leaves
STL: Spread tea leaves
FTS: Fixated tea leaves
FLS: Spread Fixated tea leaves
RTS: Rolled tea leaves
PF1: Pan-fried 1 leaves
PF2: Pan-fried 2 leaves
PF3: Pan-fried 3 leaves
DTL: Depilated tea leaves
RPL: Rotary pot first-dried leaves
BHL: Box hot air second-dried leaves
BHL70: The temperature of box hot air second-dried is 70°C
BHL90: The temperature of box hot air second-dried is 90°C
BHL110: The temperature of box hot air second-dried is 110°C
RGT: Round green tea
IRAE-HS-SPME: Infrared-assisted headspace-solid phase microextraction
GC-MS: Gas chromatography-mass spectrometry
MEV: Multiple experiment viewer
PLS-DA: Partial least-squares discriminant analysis
VIP: Variable importance projection.
