# Supplementary material for: Assessment of Variations in Round Green Tea Volatile Metabolites During Manufacturing and Effect of Second-Drying Temperature via Nontargeted Metabolomic Analysis
Source: Front Nutr. 2022 Apr 14;9:877132. doi: 10.3389/fnut.2022.877132 (PMC9047777; doi:10.3389/fnut.2022.877132)
Supplement: Supplementary file 1 [file Data_Sheet_1.PDF]

# *Supplementary Material*

## 1. Supplementary Data

Supplementary Material should be uploaded separately on submission. Please include any supplementary data, figures and/or tables. All supplementary files are deposited to FigShare for permanent storage and receive a DOI.

Supplementary material is not typeset so please ensure that all information is clearly presented, the appropriate caption is included in the file and not in the manuscript, and that the style conforms to the rest of the article. To avoid discrepancies between the published article and the supplementary material, please do not add the title, author list, affiliations or correspondence in the supplementary files.

## 2. Supplementary Figures and Tables

For more information on Supplementary Material and for details on the different file types accepted, please see here. Figures, tables, and images will be published under a Creative Commons CC-BY licence and permission must be obtained for use of copyrighted material from other sources (including re-published/adapted/modified/partial figures and images from the internet). It is the responsibility of the authors to acquire the licenses, to follow any citation instructions requested by third-party rights holders, and cover any supplementary charges.

### 2.1 Supplementary Tables

**Table S1**. List all of the volatile compounds in round green tea process.

| NO | Name | CAS | RT | $RI^A$ | $RI^B$ | FTL | STL | FTS | FLS | RTL | PF1 | PF2 | PF3 | DTL | RPL | BHL |
|---|---|---|---|---|---|---|---|---|---|---|---|---|---|---|---|---|
| 1 | Dimethyl ether | 115-10-6 | 2.15 | 738.79 | - | 7.7±0.71 | 6.6±0.62 | 7.38±0.46 | 7.75±0.58 | 8.14±0.72 | 7.41±0.47 | 8.26±0.37 | 8.49±1.21 | 7.68±0.54 | 9.03±1.56 | 8.12±0.64 |
| 2 | Butanal, 3-methyl- | 590-86-3 | 2.30 | 743.00 | 678 | 2.17±0.1 | 5.24±0.09 | 3.92±0.68 | 3.89±0.81 | 4.28±0.32 | 4.1±0.49 | 4.3±0.87 | 3.92±1.01 | 3.65±0.09 | 2.82±0.82 | 3.34±0.59 |
| 3 | Butanal, 2-methyl- | 96-17-3 | 2.40 | 745.80 | 682 | 2.78±0.1 | 7.09±0.07 | 5.49±0.99 | 5.05±1.01 | 6.14±0.06 | 5.35±0.48 | 5.73±1.42 | 5.15±1.44 | 4.95±0.05 | 3.81±0.69 | 4.63±0.85 |
| 4 | 1-Penten-3-ol | 616-25-1 | 2.61 | 751.73 | 685 | 10.29±0.54 | 11.91±1.77 | 19.9±2.57 | 19.03±0.65 | 16.16±0.94 | 16.15±1.66 | 17.28±1.37 | 22.21±5 | 22.34±2.5 | 20.41±5.63 | 11.46±1.71 |
| 5 | Pentanal | 110-62-3 | 2.80 | 757.13 | 701 | 2.13±0.15 | 2.44±0.34 | 2.92±0.41 | 2.77±0.33 | 2.42±0 | 2.87±0.15 | 3.45±0.5 | 4.52±1.43 | 5.82±0.68 | 6.03±1.14 | 5.41±1.58 |
| 6 | Dimethylphosphinic fluoride | 753-70-8 | 2.86 | 758.87 | - | 2.69±0.14 | 3.85±0.04 | 2.05±0.5 | 2.27±0.3 | 2.02±0.09 | 2.13±0.13 | 2.39±0.59 | 2.24±0.61 | 2.56±0.09 | 2.53±0.29 | 1.37±0.31 |
| 7 | Benzoyl bromide | 618-32-6 | 3.44 | 775.08 | - | 5.99±0.22 | 5.56±0.53 | 7.72±0.56 | 7.48±0.49 | 6.24±0.19 | 6.56±1.08 | 7.41±0.36 | 8.26±0.3 | 6.79±0.09 | 7.67±1.27 | 6.91±0.99 |
| 8 | 1-Butanol, 3-methyl- | 123-51-3 | 4.04 | 791.88 | 730 | 1.94±0.09 | 2.19±0.34 | 6.79±0.98 | 6.69±0.62 | 5.87±0.11 | 5.81±0.44 | 7.19±0.47 | 8.73±2.38 | 9.74±0.82 | 8.96±1.9 | 8.95±1.92 |
| 9 | 1-Butanol, 2-methyl- | 137-32-6 | 3.80 | 785.36 | 762 | 2.21±0.06 | 2.12±0.36 | 0.1±0.02 | 0.12±0.02 | 0.11±0.01 | 0.13±0.03 | 0.15±0.04 | 0.27±0.09 | 0.28±0.06 | 0.42±0.07 | 0.16±0.07 |
| 10 | Toluene | 108-88-3 | 4.11 | 793.91 | 773 | 1.71±0.51 | 1.59±0.45 | 1.89±0.28 | 2.39±0.33 | 2.07±0.08 | 1.77±0.56 | 1.69±0.28 | 1.93±0.56 | 2.13±0.3 | 2.05±0.09 | 1.4±0.4 |
| 11 | 1-Pentanol | 71-41-0 | 4.15 | 795.14 | 779 | 7.81±0.27 | 8.22±0.88 | 6.82±0.97 | 6.73±0.57 | 5.89±0.12 | 5.84±0.44 | 7.21±0.45 | 8.73±2.41 | 9.77±0.83 | 8.98±1.88 | 8.98±1.92 |
| 12 | Cyclobutanemethanol | 4415-82-1 | 4.24 | 797.56 | - | 3.58±0.43 | 4.7±0.45 | 11.3±1.04 | 11.45±0.44 | 9.18±0.89 | 9.42±0.87 | 10.66±0.25 | 12.57±2.43 | 11.97±1.34 | 11.37±3.15 | 6.71±0.85 |
| 13 | Furan, 2-methoxy- | 25414-22-6 | 4.90 | 816.20 | - | 3.23±0.07 | 3.41±0.13 | 5.16±0.87 | 4.31±0.41 | 3.48±0.15 | 3.32±0.43 | 4.14±0.44 | 3.69±0.84 | 3.74±0.11 | 3.39±0.71 | 10.76±1.3 |
| 14 | Hexanal | 66-25-1 | 4.92 | 816.87 | 800 | 11.55±0.66 | 15.64±1.19 | 9.83±1.11 | 9.18±1.05 | 6.65±0.27 | 8.17±0.47 | 9.03±1.22 | 12.22±3.43 | 14.98±2.34 | 15.77±3.22 | 10.71±2.47 |
| 15 | Cyclopentane, (2-methylpropyl)- | 3788-32-7 | 6.44 | 859.61 | - | 1.51±0.14 | 3.5±0.42 | 0.78±0.06 | 0.84±0.09 | 0.67±0.05 | 0.71±0.03 | 0.91±0.11 | 1.15±0.28 | 1.16±0.18 | 1.43±0.32 | 0.87±0.2 |
| 16 | 3-Hexen-1-ol, (E)- | 928-97-2 | 6.54 | 862.43 | 844 | 82.86±2.69 | 76.57±6.53 | 12.06±1.16 | 10.52±0.41 | 8.91±0.96 | 6.5±0.54 | 6.86±0.3 | 9.03±1.75 | 8.49±1.17 | 7.73±2.14 | 6.68±0.72 |
| 17 | 1-Hexanol | 111-27-3 | 6.96 | 874.16 | 879 | 58.49±0.86 | 62.18±5.67 | 10.83±1.58 | 8.78±0.51 | 7.32±0.52 | 6.39±0.53 | 7.75±0.54 | 8.53±1.77 | 8.35±0.95 | 7.07±1.91 | 5.43±0.63 |
| 18 | 1,3-Cyclohexadiene, 5-ethyl- | 40085-08-3 | 7.22 | 881.60 | - | 3.05±0.2 | 3.94±0.17 | 0.81±0.15 | 1.24±0.16 | 1.43±0.16 | 1.31±0.13 | 1.72±0.51 | 1.01±0.29 | 0.94±0.03 | 0.91±0.1 | 0.62±0.16 |
| 19 | Styrene | 100-42-5 | 7.55 | 890.82 | 890 | 5.43±2.76 | 4.14±2.41 | 2.51±0.28 | 1.91±0.23 | 1.89±0.14 | 3.61±3.77 | 1.99±0.44 | 2.37±0.67 | 3.03±0.87 | 1.88±0.26 | 0.78±0.09 |
| 20 | 2-Heptanone | 110-43-0 | 7.61 | 892.58 | 895 | 2.92±0.19 | 3.49±0.18 | 2.5±0.43 | 2.27±0.2 | 1.9±0.07 | 1.99±0.18 | 2.22±0.37 | 2.36±0.6 | 2.4±0.16 | 2.06±0.43 | 1.63±0.3 |
| 21 | Heptanal | 111-71-7 | 7.93 | 901.59 | 901 | 8.89±0.6 | 11.21±0.6 | 14.78±1.68 | 16.24±1.42 | 12.23±0.66 | 12.87±0.51 | 15.29±1.57 | 18.77±4.74 | 21.58±2.74 | 20.37±4.88 | 11.93±1.86 |
| 22 | Oxime-, methoxy-phenyl-_ | 1000222-86-6 | 8.33 | 912.72 | - | 13.9±3.01 | 16.6±7.87 | 24.44±2.72 | 24.42±0.43 | 23.56±2.64 | 23.39±5.48 | 24.16±0.42 | 26.79±3.56 | 24.02±1.4 | 25.16±3.17 | 23.05±4.16 |
| 23 | Benzene, 1-methoxy-4-methyl- | 104-93-8 | 9.00 | 931.78 | - | 1.97±0.02 | 2.4±0.02 | 1.66±0.23 | 1.64±0.08 | 1.52±0.15 | 1.31±0.14 | 1.54±0.14 | 1.16±0.22 | 0.93±0.02 | 0.65±0.2 | 1.01±0.05 |

| | | | | | | | | | | | | | | | | |
|---|---|---|---|---|---|---|---|---|---|---|---|---|---|---|---|---|
| 24 | (S)-(+)-5-Methyl-1-heptanol | 57803-73-3 | 9.70 | 951.30 | - | 1.53±0.26 | 1.84±0.25 | 3.15±0.5 | 3.43±0.07 | 3.05±0.07 | 3.98±0.54 | 6.09±1.53 | 5.67±2.02 | 5.73±0.67 | 8.27±1.07 | 9.77±2.84 |
| 25 | Benzaldehyde | 100-52-7 | 9.78 | 953.53 | 961 | 281.81±9.76 | 260.89±16.5 | 75.25±4.1 | 74.12±5.27 | 71.24±7.06 | 57.18±2.23 | 57.7±2.36 | 58.29±6.59 | 44.57±4.05 | 45.57±10.89 | 33.69±3.35 |
| 26 | 1-Heptanol | 111-70-6 | 10.18 | 964.81 | 969 | 37.22±2.89 | 38.22±2.66 | 4.76±0.57 | 3.39±0.15 | 2.67±0.18 | 2.38±0.12 | 2.68±0.24 | 3.05±0.51 | 2.78±0.35 | 2.49±0.61 | 2.33±0.23 |
| 27 | 1-Octen-3-ol | 3391-86-4 | 10.47 | 973.04 | 976 | 26±1.07 | 31.58±1.28 | 22.42±2.8 | 23.13±1.69 | 25.01±0.34 | 20.7±1.24 | 32.44±5.37 | 33.57±12.36 | 29.34±0.69 | 30.53±5.83 | 36.04±7.08 |
| 28 | Azetidine, 3-methyl-3-phenyl- | 5961-33-1 | 10.50 | 973.84 | - | 1.66±0.74 | 1.32±0.64 | 0.52±0.06 | 0.55±0.13 | 0.73±0.23 | 0.4±0.06 | 0.42±0.06 | 0.68±0.12 | 0.57±0.18 | 0.37±0.05 | 0.21±0.03 |
| 29 | Pentanoic acid, 2-methyl-, anhydride | 63169-61-9 | 10.65 | 978.02 | - | 9.42±0.3 | 12.74±0.53 | 22.47±1.91 | 23.19±2.83 | 20.17±1.74 | 21.45±0.68 | 28.05±1.94 | 30.97±8.85 | 27.37±0.27 | 25.86±2.96 | 25.41±3.4 |
| 30 | Vinyl butyrate | 123-20-6 | 10.71 | 979.96 | - | 5.52±0.43 | 5.13±0.2 | 3.13±0.28 | 2.93±0.22 | 2.72±0.05 | 2.55±0.07 | 2.96±0.16 | 3.73±1.03 | 3.36±0.23 | 3.1±0.57 | 2.9±0.41 |
| 31 | 5-Hepten-2-one, 6-methyl- | 110-93-0 | 10.72 | 980.07 | 988 | 10.27±0.85 | 10.15±0.97 | 7.83±0.95 | 7.39±0.41 | 6.77±0.07 | 6.1±0.23 | 7.55±0.73 | 9.26±2.48 | 8.34±0.81 | 7.52±1.54 | 6.72±0.97 |
| 32 | Bicyclo[3.1.1]heptane, 6,6-dimethyl-2-methylene-, (1S)- | 18172-67-3 | 10.84 | 983.57 | - | 98.17±2.78 | 97.27±7.4 | 15.2±1.46 | 16.75±1.06 | 14.17±0.3 | 11.21±1.18 | 11±0.23 | 12.8±1.62 | 11.89±1.78 | 9.98±1.76 | 9.04±0.78 |
| 33 | Hexanoic acid, ethyl ester | 123-66-0 | 11.14 | 991.94 | 996 | 9.73±0.6 | 12.1±0.65 | 2.49±0.3 | 2.91±0.22 | 2.03±0.21 | 1.74±0.24 | 2.4±0.38 | 3.54±0.96 | 3.22±0.44 | 2.61±0.44 | 2.42±0.12 |
| 34 | cis-2-(2-Pentenyl)furan | 70424-13-4 | 11.16 | 992.42 | - | 0.99±0.14 | 1.39±0.08 | 1.61±0.2 | 1.69±0.14 | 1.61±0.05 | 1.53±0.12 | 1.57±0.18 | 1.52±0.26 | 1.37±0.15 | 1.17±0.25 | 0.68±0.08 |
| 35 | Octanal | 124-13-0 | 11.21 | 993.81 | 1001 | 1.46±0.27 | 1.81±0.58 | 1.88±0.05 | 1.93±0.2 | 1.53±0.05 | 1.56±0.22 | 1.64±0.55 | 1.87±0.31 | 1.7±0.54 | 1.83±0.87 | 1.11±0.15 |
| 36 | 4-Hexen-1-ol, (4E)-, acetate | 1000352-71-9 | 11.43 | 999.99 | - | 169.89±7.35 | 23.54±1.88 | 3±0.25 | 3.53±0.36 | 3.14±0.06 | 3.59±0.32 | 3.97±0.34 | 4.76±0.66 | 4.03±0.72 | 4.83±0.88 | 2.03±0.58 |
| 37 | Furan, 2-propyl- | 4229-91-8 | 11.44 | 1000.31 | - | 11.69±0.63 | 20.32±2.67 | 33.53±1.16 | 42.63±6.67 | 39.85±2.89 | 45.48±4.77 | 52.16±4.34 | 61.44±9.14 | 52.1±8.94 | 64.24±11.61 | 28.44±5.66 |
| 38 | Acetic acid, hexyl ester | 142-92-7 | 11.55 | 1003.56 | 1008 | 0.74±0.03 | 0.12±0.02 | 0.05±0.02 | 0.05±0.01 | 0.04±0 | 0.04±0.02 | 0.05±0.03 | 0.03±0.01 | 0.08±0.01 | 0.09±0.03 | 0.04±0.01 |
| 39 | Ethanone, 1-(3-hydroxyphenyl)- | 121-71-1 | 11.61 | 1005.14 | - | 3.52±0.11 | 3.75±0.27 | 0.78±0.17 | 0.76±0.03 | 0.58±0 | 0.65±0.11 | 0.63±0.02 | 0.55±0.1 | 0.47±0.05 | 0.37±0.06 | 0.52±0.02 |
| 40 | 2-Hexen-1-ol, acetate, (E)- | 2497-18-9 | 11.29 | 996.03 | 1007 | 1.79±0.12 | 0.16±0.02 | 1.13±0.03 | 1.14±0.1 | 0.88±0.03 | 0.91±0.11 | 1.05±0.13 | 1.14±0.18 | 1.1±0.39 | 1.09±0.49 | 0.7±0.1 |
| 41 | o-Cymene | 527-84-4 | 11.87 | 1012.39 | 1020 | 3.09±0.1 | 3.28±0.22 | 1.37±0.24 | 1.22±0.08 | 0.94±0.05 | 0.84±0.09 | 0.82±0.13 | 0.79±0.14 | 0.8±0.12 | 0.69±0.03 | 0.84±0.15 |
| 42 | D-Limonene | 5989-27-5 | 11.99 | 1015.85 | 1029 | 14.11±0.34 | 16.01±0.62 | 3.92±0.8 | 4.43±0.32 | 3.99±1.03 | 3.65±1.76 | 3.87±2.28 | 3.46±0.91 | 4.29±1.97 | 3.54±1.11 | 4.89±1.24 |
| 43 | 1-Hexanol, 2-ethyl- | 104-76-7 | 12.05 | 1017.57 | 1028 | 21.99±2.3 | 20.56±2.19 | 5.84±0.91 | 7.64±0.33 | 6.55±0.77 | 6±0.47 | 8.51±2.04 | 8.18±2.03 | 8.14±0.13 | 7±1.22 | 8.85±1.08 |
| 44 | Cyclohexanone, 2,2,6-trimethyl- | 2408-37-9 | 12.13 | 1019.82 | 1023 | 93.33±74.88 | 6.33±0.4 | 6.44±1.11 | 6.55±0.62 | 6±0.12 | 5.98±0.5 | 6.89±0.84 | 7.37±2.05 | 7.31±0.57 | 6.74±1.58 | 4.72±0.9 |
| 45 | Benzyl alcohol | 100-51-6 | 12.24 | 1022.79 | 1033 | 80.03±1.71 | 78.75±7.42 | 23.3±1.54 | 15.61±12.6 | 20.83±3 | 17.48±0.97 | 21.23±2.74 | 21.23±0.03 | 15.92±0.18 | 16.45±3.86 | 15.17±1.37 |
| 46 | (1R)-2,6,6-Trimethylbicyclo[3.1.1]hept-2-ene | 7785-70-8 | 12.34 | 1025.82 | - | 23.5±1.48 | 24.22±1.47 | 2.66±0.26 | 3.04±0.17 | 2.5±0.07 | 2.14±0.25 | 2.07±0.12 | 1.84±0.16 | 1.5±0.09 | 1.48±0.37 | 1.62±0.22 |
| 47 | 3-Octen-2-one | 1669-44-9 | 12.37 | 1026.58 | 1037 | 5.64±0.23 | 6.49±0.46 | 2.51±0.16 | 2.62±0.12 | 2.13±0.23 | 2.27±0.06 | 2.5±0.21 | 2.94±0.51 | 2.38±0.36 | 2.23±0.53 | 1.64±0.1 |
| 48 | Benzeneacetaldehyde | 122-78-1 | 12.48 | 1029.57 | 1043 | 5.66±0.96 | 11.06±0.93 | 16.05±2.44 | 20.33±1.88 | 20.42±0.09 | 18.79±1.31 | 18.83±3.48 | 14.2±0.7 | 13.4±0.48 | 15±3.59 | 13.73±2.86 |
| 49 | 3-Carene | 13466-78-9 | 12.66 | 1034.73 | 1018 | 15.1±0.52 | 14.63±0.68 | 2.97±0.24 | 2.86±0.16 | 2.42±0.01 | 1.98±0.11 | 1.74±0.06 | 1.6±0.15 | 1.39±0.11 | 1.34±0.33 | 2.99±0.13 |

| | | | | | | | | | | | | | | | | |
|---|---|---|---|---|---|---|---|---|---|---|---|---|---|---|---|---|
| 50 | Isophorone | 78-59-1 | 12.97 | 1043.59 | 1124 | 12.63±0.41 | 10.36±1.67 | 9.04±1.03 | 9.28±0.41 | 8.38±0.15 | 8.25±0.59 | 9.19±0.88 | 10.17±1.96 | 9.82±1.52 | 9.65±2.29 | 7.09±1.12 |
| 51 | Acetophenone | 98-86-2 | 13.19 | 1049.54 | 1065 | 5.03±0.44 | 4.75±0.5 | 2.54±1.14 | 2.86±1.11 | 2.35±1.23 | 1.34±0.16 | 1.43±0.16 | 1.51±0.52 | 1.33±0.54 | 1.96±0.47 | 1.13±0.48 |
| 52 | Ethanone, 1-(1-cyclohexen-1-yl)- | 932-66-1 | 13.38 | 1054.98 | - | 51.65±1.6 | 57.36±2.81 | 22.8±0.56 | 26.44±2.58 | 24.46±3.52 | 23.81±0.77 | 26.34±1.91 | 27.94±3.02 | 22.92±3.16 | 22.17±4.92 | 12.14±0.55 |
| 53 | Formic acid, octyl ester | 112-32-3 | 13.42 | 1056.03 | 1117 | 33.89±0.76 | 39.39±0.56 | 14.29±1.15 | 15.38±0.7 | 13.28±1.04 | 12.29±0.57 | 14.01±0.67 | 12.67±1.78 | 11.48±0.81 | 10.42±2.66 | 9.93±0.83 |
| 54 | Ethanone, 1-(4-methylphenyl)- | 122-00-9 | 13.65 | 1062.59 | 1183 | 0.71±0.29 | 0.77±0.17 | 0.36±0.07 | 0.42±0.03 | 0.38±0 | 0.33±0.04 | 0.35±0.01 | 0.26±0.02 | 0.26±0.01 | 0.22±0.07 | 0.25±0.03 |
| 55 | Benzene, 4-ethenyl-1,2-dimethyl- | 27831-13-6 | 13.93 | 1070.36 | - | 1.91±0.07 | 2.24±0.3 | 1.88±0.22 | 1.77±0.07 | 1.49±0.11 | 1.29±0.1 | 1.22±0.12 | 0.91±0.08 | 0.81±0.02 | 0.66±0.24 | 0.87±0.04 |
| 56 | Ethyl 2-(5-methyl-5-vinyltetrahydrofuran-2-yl)propan-2-yl carbonate | 1000373-80-3 | 13.91 | 1070.07 | - | 84.51±1.74 | 96.87±4.38 | 12.5±0.81 | 14.26±0.57 | 11.8±1.43 | 8.31±0.41 | 8.57±0.29 | 8.34±1.12 | 8±1.05 | 7.14±2.04 | 6.97±0.33 |
| 57 | Benzoic acid, hydrazide | 613-94-5 | 14.06 | 1074.03 | - | 3.17±2.08 | 3.23±2.11 | 1.1±0.14 | 1.14±0.13 | 1.09±0.18 | 1.06±0.09 | 1.05±0.08 | 1.06±0.07 | 0.7±0.04 | 0.67±0.21 | 0.34±0.03 |
| 58 | Linalool | 78-70-6 | 14.51 | 1086.86 | | 336.99±135.22 | 433.58±15.04 | 12.23±1.08 | 11.97±0.85 | 9.65±0.99 | 10.04±0.53 | 11.13±0.88 | 12.71±1.31 | 11.5±1.35 | 11.86±3.08 | 7.41±0.76 |
| 59 | 1,5,7-Octatrien-3-ol, 3,7-dimethyl- | 29957-43-5 | 14.46 | 1085.51 | 1100 | 17.94±3.18 | 23.48±0.5 | 31.08±2.06 | 34.83±0.92 | 27.94±3.72 | 25.54±1.82 | 27.67±0.99 | 24.31±2.78 | 20.87±1.35 | 18.9±5.19 | 29.64±1.4 |
| 60 | Phenylethyl Alcohol | ######## | 14.68 | 1091.56 | 1116 | 74.47±1.22 | 72.62±5.08 | 22.14±1.91 | 23.8±2.12 | 21.93±2.9 | 18.24±1.19 | 20.62±2.42 | 19.26±0.39 | 15.31±0.22 | 14.87±3.48 | 15.41±1.58 |
| 61 | (E)-4,8-Dimethylnona-1,3,7-triene | 19945-61-0 | 14.77 | 1094.02 | 1116.5 | 14.39±1.08 | 167.01±4.75 | 4.62±0.52 | 4.82±0.32 | 4.18±0.31 | 3.3±0.22 | 3.23±0.36 | 3.3±0.78 | 2.9±0.25 | 2.15±0.31 | 1.95±0.08 |
| 62 | 1,3,8-p-Menthatriene | 18368-95-1 | 14.93 | 1098.73 | 1118.7 | 1.76±0.11 | 1.73±0.02 | 0.35±0.05 | 0.4±0.03 | 0.34±0.04 | 0.28±0.03 | 0.27±0.02 | 0.16±0.08 | 0.14±0.01 | 0.15±0.05 | 0.25±0.01 |
| 63 | 2,4,6-Octatriene, 2,6-dimethyl- | 673-84-7 | 15.14 | 1104.44 | 1113 | 2.58±0.35 | 39.76±32.04 | 0.48±0.05 | 0.47±0.03 | 0.36±0 | 0.31±0.02 | 0.28±0.01 | 0.27±0.02 | 0.24±0.02 | 0.23±0.05 | 0.24±0.02 |
| 64 | 2,6-Dimethyl-1,3,5,7-octatetraene, E,E- | 460-01-5 | 15.18 | 1105.76 | 1130 | 4.94±0.17 | 5.23±0.15 | 1.2±0.1 | 1.24±0.06 | 1.2±0.07 | 0.88±0.06 | 0.84±0.05 | 0.63±0.05 | 0.48±0.01 | 0.44±0.11 | 0.78±0.02 |
| 65 | n-Pentadecanol | 629-76-5 | 15.24 | 1107.37 | - | 3.66±0.21 | 3.96±0.6 | 0.58±0.03 | 0.46±0.05 | 0.39±0.04 | 0.36±0.05 | 0.28±0.02 | 0.35±0.09 | 0.26±0.06 | 0.22±0.04 | 0.19±0.02 |
| 66 | Benzyl nitrile | 140-29-4 | 15.44 | 1112.98 | 1143 | 7.65±0.2 | 14.87±0.06 | 4.76±0.51 | 4.64±0.45 | 3.64±0.53 | 3.3±0.26 | 3.28±0.32 | 2.74±0.01 | 2.16±0.07 | 2.15±0.58 | 3.57±0.15 |
| 67 | trans-3-Nonen-2-one | 18402-83-0 | 15.48 | 1114.06 | - | 1.42±0.04 | 1.47±0.02 | 0.77±0.06 | 0.96±0.07 | 0.87±0.11 | 0.85±0.05 | 0.85±0.03 | 0.77±0.09 | 0.58±0.05 | 0.56±0.15 | 0.38±0.02 |
| 68 | 2,4,6-Octatriene, 2,6-dimethyl-, (E,Z)- | 7216-56-0 | 15.53 | 1115.45 | 1131 | 2.31±0.06 | 2.38±0.13 | 0.54±0.06 | 0.42±0.05 | 0.45±0.1 | 0.23±0.02 | 0.2±0.01 | 0.17±0 | 0.15±0.02 | 0.16±0.04 | 0.24±0.03 |
| 69 | (R,S)-5-Ethyl-6-methyl-3E-hepten-2-one | 57283-79-1 | 15.68 | 1119.87 | 1143.9 | 5.01±0.25 | 7.41±0.29 | 2.98±0.27 | 3.18±0.09 | 2.55±0.02 | 2.74±0.1 | 2.89±0.21 | 3.02±0.44 | 2.51±0.28 | 2.19±0.47 | 1.65±0.15 |
| 70 | 1,12-Dodecanediol | 5675-51-4 | 16.28 | 1136.75 | - | 4.25±0.22 | 5.3±0.2 | 4.15±0.19 | 5.8±0.35 | 4.66±0.69 | 3.73±0.27 | 4.24±0.45 | 3.4±0.06 | 2.94±0.17 | 2.65±0.76 | 2.76±0.2 |
| 71 | Undecane, 3-methyl- | 1002-43-3 | 16.38 | 1139.47 | 1171 | 5.7±0.68 | 6.11±0.72 | 1.51±0.07 | 1.79±0.1 | 2.19±0.32 | 1.28±0.1 | 1.51±0.11 | 1.82±0.33 | 1.59±0.16 | 0.95±0.11 | 0.9±0.04 |
| 72 | Benzoic acid, ethyl ester | 93-89-0 | 16.40 | 1140.04 | 1170 | 2.38±0.11 | 2.21±0.08 | 0.73±0.01 | 0.75±0.04 | 3.24±2.52 | 1.12±0.76 | 0.67±0.12 | 0.43±0.1 | 0.46±0.02 | 0.41±0.19 | 0.36±0.1 |
| 73 | Benzaldehyde, 2,5-dimethyl- | 5779-94-2 | 16.50 | 1142.79 | 1208 | 1.49±0.07 | 1.72±0.1 | 1.63±0.22 | 1.97±0.18 | 1.85±0.15 | 1.96±0.27 | 1.9±0.14 | 1.4±0.02 | 1.13±0.07 | 1.2±0.33 | 0.82±0.05 |
| 74 | 1-Nonanol | 143-08-8 | 16.52 | 1143.40 | 1186 | 76.33±2.16 | 77.87±0.59 | 1.32±0.09 | 1.52±0.03 | 1.25±0.16 | 0.98±0.08 | 1.11±0.15 | 1.01±0.05 | 0.91±0.07 | 0.62±0.22 | 0.85±0.05 |
| 75 | Naphthalene | 91-20-3 | 16.70 | 1148.61 | 1191 | 1.24±0.1 | 1.12±0.06 | 0.43±0.02 | 0.45±0.05 | 0.44±0.07 | 0.38±0.01 | 0.4±0.01 | 0.37±0.03 | 0.33±0.02 | 0.25±0.12 | 0.26±0.06 |

| | | | | | | | | | | | | | | | | |
|---|---|---|---|---|---|---|---|---|---|---|---|---|---|---|---|---|
| 76 | Butanoic acid, 3-hexenyl ester, (Z)- | 16491-36-4 | 16.97 | 1156.15 | 1186 | 84.47±2.52 | 20.34±1.5 | 1.64±0.25 | 1.92±0.08 | 2.02±0.51 | 1.72±0.54 | 1.8±0.51 | 0.97±0.18 | 0.76±0.11 | 0.56±0.12 | 0.83±0.17 |
| 77 | Butanoic acid, 2-hexenyl ester, (Z)- | 56922-77-1 | 17.17 | 1161.67 | - | 4.58±0.24 | 1.96±0.38 | 2.64±0.2 | 2.67±0.11 | 1.94±0.2 | 1.01±0.02 | 0.82±0.08 | 4.97±3.86 | 0.77±0.11 | 4.95±0.18 | 4±0.26 |
| 78 | Methyl salicylate | 119-36-8 | 17.11 | 1160.06 | 1187 | 527.58±28.95 | 559.07±11.97 | 58.23±11.65 | 52.28±5.84 | 52.72±9.06 | 32.03±1.74 | 28.02±0.97 | 23.29±1.53 | 19.17±2.18 | 18.15±4.48 | 16.54±0.34 |
| 79 | Dodecane | 112-40-3 | 17.25 | 1164.04 | - | 43.57±7.2 | 41.7±7.82 | 13.3±0.73 | 15.81±1.07 | 16.36±3.63 | 11.64±0.76 | 12.45±1.46 | 16.66±3.25 | 13.44±0.76 | 7.42±0.35 | 6.25±0.32 |
| 80 | 1,3-Cyclohexadiene-1-carboxaldehyde, 2,6,6-trimethyl- | 116-26-7 | 17.24 | 1163.71 | 1197 | 185.13±9.03 | 195.97±5.63 | 4.16±0.9 | 4.12±0.1 | 3.04±0.51 | 3.5±0.87 | 3.61±0.18 | 2.52±0.17 | 1.69±0.03 | 1.62±0.48 | 1.93±0.05 |
| 81 | Decanal | 112-31-2 | 17.44 | 1169.34 | 1195 | 5.2±0.06 | 10.44±0.29 | 1.5±0.05 | 1.67±0.16 | 1.36±0.18 | 1.43±0.05 | 1.66±0.13 | 1.56±0.23 | 1.39±0.12 | 1.47±0.42 | 1.31±0.14 |
| 82 | Formamide, N-phenyl- | 103-70-8 | 17.65 | 1175.34 | - | 185.11±9.03 | 195.91±5.6 | 1.12±0.18 | 1.15±0.07 | 0.97±0.09 | 0.49±0.06 | 0.48±0.01 | 0.38±0.01 | 0.33±0.03 | 0.36±0.07 | 0.35±0.04 |
| 83 | 1-Cyclohexene-1-carboxaldehyde, 2,6,6-trimethyl- | 432-25-7 | 17.86 | 1181.05 | 1214 | 16.71±0.52 | 17.85±0.56 | 16.57±1.38 | 17.98±0.85 | 15.17±1.07 | 15.61±0.82 | 16.72±0.77 | 16.7±2.41 | 14.99±1.59 | 14.01±3.37 | 10.07±0.76 |
| 84 | 2,6-Octadien-1-ol, 3,7-dimethyl-, (Z)- | 106-25-2 | 18.72 | 1205.35 | 1229 | 43.68±1.29 | 47.43±1.36 | 90.85±5.95 | 110.29±10.26 | 109.11±12.27 | 91.17±6.59 | 86.86±5.09 | 79.46±1.32 | 65.94±7.22 | 68.21±18.45 | 61.51±3.29 |
| 85 | n-Valeric acid cis-3-hexenyl ester | 35852-46-1 | 18.20 | 1190.62 | 1235 | 53.82±2.03 | 15.14±0.85 | 11.3±0.63 | 12.6±0.55 | 12.28±1.28 | 7.58±0.03 | 6.9±0.48 | 6.89±1.27 | 5.9±0.64 | 4.51±0.97 | 4.02±0.11 |
| 86 | Valeric anhydride | 2082-59-9 | 18.31 | 1193.72 | - | 5.54±0.22 | 3.24±0.16 | 1.39±0.16 | 1.63±0.1 | 1.38±0.13 | 1.06±0.05 | 1.05±0.06 | 1.27±0.17 | 1.12±0.16 | 0.87±0.3 | 0.65±0.02 |
| 87 | trans-2-Hexenyl isovalerate | 68698-59-9 | 18.37 | 1195.46 | 1244.9 | 3.54±0.19 | 2.3±0.06 | 1.75±0.11 | 2.52±0.12 | 1.99±0.22 | 1.15±0.02 | 1.15±0.04 | 1.13±0.17 | 0.92±0.11 | 0.73±0.17 | 0.51±0.34 |
| 88 | 3H-Indazol-3-one, 1,2-dihydro-1-methyl- | 1006-19-5 | 18.38 | 1195.77 | - | 1.15±0.04 | 0.94±0.04 | 0.12±0.02 | 0.1±0.01 | 0.1±0.01 | 0.49±0.36 | 0.28±0.36 | 0.08±0.01 | 0.38±0.31 | 0.32±0.25 | 0.1±0.05 |
| 89 | 1-Tetradecanol | 112-72-1 | 18.55 | 1200.54 | - | 9.64±0.33 | 75.29±112.03 | 6.51±9.54 | 9.16±13.86 | 10.63±9.3 | 13.25±10.72 | 6.87±10.12 | 1.18±0.22 | 1±0.06 | 13.7±3.71 | 8.55±6.89 |
| 90 | Geraniol | 106-24-1 | 18.93 | 1211.18 | 1257 | 552.91±26.63 | 692.45±167.79 | 91.11±5.94 | 110.32±10.25 | 109.27±12.11 | 91.12±6.61 | 86.96±4.99 | 79.6±1 | 66.08±7.21 | 44.74±41.92 | 61.56±3.34 |
| 91 | Benzoic acid, 2-hydroxy-, ethyl ester | 118-61-6 | 19.28 | 1221.29 | 1267 | 14.51±2.06 | 28.76±2.9 | 0.76±0.66 | 0.48±0.38 | 0.59±0.07 | 0.22±0.33 | 0.17±0.24 | 0.2±0.17 | 0.27±0.02 | 0.29±0.04 | 0.26±0.01 |
| 92 | Citral | 5392-40-5 | 19.14 | 1217.33 | 1237 | 31.44±0.81 | 316.26±493.88 | 5.65±0.49 | 6.35±0.6 | 63.2±58.18 | 35.22±53.06 | 61.29±49.24 | 41.44±37.26 | 3.46±0.23 | 3.72±0.9 | 61.57±3.29 |
| 93 | 1-Decanol | 112-30-1 | 19.42 | 1225.05 | 1272 | 10.2±0.23 | 9.1±0.64 | 0.91±0.45 | 1.19±0.12 | 1.08±0.06 | 0.83±0.23 | 0.64±0.21 | 0.64±0.08 | 0.59±0.12 | 0.52±0.23 | 1.02±0.99 |
| 94 | 2-Aminosuccinonitrile | 5615-94-1 | 19.85 | 1237.21 | - | 6.87±1.03 | 6.07±1.12 | 0.81±0.25 | 1.09±0.03 | 0.94±0.38 | 0.85±0.01 | 0.78±0.08 | 0.56±0.22 | 0.24±0.03 | 0.19±0.08 | 0.26±0.11 |
| 95 | n-Tridecan-1-ol | 112-70-9 | 19.85 | 1237.24 | - | 5.36±1.09 | 5.29±2.63 | 1.02±0.16 | 1.15±0.07 | 1.22±0.31 | 0.87±0.03 | 0.9±0.07 | 0.66±0.32 | 0.25±0 | 0.19±0.03 | 0.28±0.25 |
| 96 | 2-Undecanone | 112-12-9 | 19.90 | 1238.71 | 1291 | 8.55±0.67 | 6.78±0.16 | 2.28±0.17 | 2.11±0.18 | 1.5±0.18 | 1.52±0.63 | 1.51±0.41 | 1.19±0.46 | 0.68±0.03 | 0.56±0.13 | 0.51±0.3 |
| 97 | Indole | 120-72-9 | 19.88 | 1238.04 | 1294 | 60.61±7.05 | 43.52±1.49 | 24.51±2.36 | 20.67±2.51 | 18.46±2.28 | 18.04±1.25 | 15.49±1.36 | 14.52±0.62 | 11.97±0.5 | 12.33±2.72 | 11.98±1.36 |
| 98 | Tridecane | 629-50-5 | 20.06 | 1243.01 | - | 50.24±2.14 | 40.65±0.67 | 8.84±0.26 | 10.77±0.92 | 9.09±2.06 | 7.11±0.12 | 6.81±0.78 | 11.8±2.71 | 8.32±1.45 | 4.82±1.23 | 4.18±1.69 |
| 99 | Nonane, 5-butyl- | 17312-63-9 | 20.20 | 1246.98 | - | 5.01±0.17 | 4.25±0.11 | 1±0.06 | 1.15±0.07 | 1.13±0.2 | 0.78±0.03 | 0.8±0.14 | 0.54±0.03 | 0.66±0.13 | 0.34±0.17 | 0.48±0.13 |
| 100 | Naphthalene, 2-methyl- | 91-57-6 | 20.31 | 1250.08 | 1315 | 4.36±0.27 | 3.86±0.21 | 0.99±0.14 | 1.26±0.14 | 1.16±0.2 | 0.99±0.07 | 1.02±0.11 | 1.06±0.1 | 0.67±0.24 | 0.71±0.18 | 0.75±0.09 |
| 101 | trans-Geranic acid methyl ester | 1189-09-9 | 20.72 | 1261.57 | 1321 | 13.84±0.53 | 7.93±0.16 | 1.4±0.09 | 1.41±0.11 | 1.36±0.22 | 0.94±0.06 | 0.79±0.03 | 0.78±0.07 | 0.57±0.07 | 0.48±0.13 | 0.5±0.05 |

| | | | | | | | | | | | | | | | | |
|---|---|---|---|---|---|---|---|---|---|---|---|---|---|---|---|---|
| 102 | (E)-Hex-3-enyl (E)-2-methylbut-2-enoate | 1000373-74-1 | 20.73 | 1262.03 | - | 10.73±0.3 | 4.98±0.02 | 1.46±0.17 | 1.81±0.13 | 1.88±0.27 | 1.03±0.19 | 0.99±0.11 | 0.95±0.22 | 0.66±0.09 | 0.64±0.18 | 0.55±0.25 |
| 103 | 1,2-Ethanediol, monobenzoate | 94-33-7 | 20.80 | 1264.09 | - | 1.46±0.1 | 1.35±0.71 | 2.46±0.22 | 2.52±0.17 | 2.33±0.57 | 1.88±0.11 | 2.17±0.44 | 1.77±0.19 | 1.11±0.09 | 0.91±0.18 | 1.19±0.28 |
| 104 | Dodecane, 1,1'-oxybis- | 4542-57-8 | 21.02 | 1270.02 | - | 10.37±1.78 | 9.78±1.29 | 2.32±0.16 | 2.34±0.26 | 2.69±0.42 | 1.98±0.19 | 1.94±0.3 | 2.53±0.65 | 1.87±0.02 | 1±0.28 | 2.15±1.76 |
| 105 | Nonadecane, 9-methyl- | 13287-24-6 | 21.11 | 1272.74 | - | 13.46±0.52 | 11.49±0.79 | 2.61±0.16 | 2.77±0.2 | 2.97±0.5 | 1.94±0.23 | 2.17±0.23 | 2.52±0.53 | 1.83±0.1 | 1.1±0.31 | 1.33±0.23 |
| 106 | Benzoic acid, 2-methoxy-, methyl ester | 606-45-1 | 21.15 | 1273.72 | 1295 | 1.92±0.12 | 0.81±0.06 | 0.45±0.06 | 0.47±0.06 | 0.52±0.06 | 0.45±0.05 | 0.39±0.05 | 0.17±0.16 | 0.25±0 | 0.14±0.13 | 0.17±0.14 |
| 107 | Heptylcyclohexane | 5617-41-4 | 21.17 | 1274.44 | - | 7.49±0.65 | 6.97±0.43 | 1.87±0.12 | 2.04±0.11 | 2.02±0.38 | 1.49±0.02 | 1.69±0.17 | 1.91±0.29 | 1.41±0.15 | 0.88±0.23 | 0.98±0.12 |
| 108 | Butane, 2,2-dimethyl- | 75-83-2 | 21.31 | 1278.30 | - | 0.98±0.17 | 0.8±0.31 | 0.18±0.01 | 0.25±0.08 | 0.2±0.05 | 0.16±0.06 | 0.18±0.04 | 0.24±0.08 | 0.15±0.03 | 0.07±0.02 | 0.17±0.09 |
| 109 | (1S,4S,4aS)-1-Isopropyl-4,7-dimethyl-1,2,3,4,4a,5-hexahydronaphthalene | 267665-20-3 | 21.42 | 1281.52 | - | 8.53±0.35 | 8.14±0.36 | 5.3±0.51 | 6.04±0.45 | 5.37±0.49 | 5.32±0.77 | 4.79±0.34 | 4.1±0.07 | 3.05±0.08 | 2.84±0.81 | 2.88±0.13 |
| 110 | Tridecane, 5-methyl- | 25117-31-1 | 21.48 | 1283.18 | 1355 | 5.64±0.95 | 5.41±0.16 | 1.23±0.06 | 1.41±0.08 | 1.48±0.28 | 0.97±0.1 | 1.15±0.12 | 1.33±0.22 | 0.92±0.1 | 0.61±0.2 | 0.9±0.24 |
| 111 | 1-Aminocyclopentanecarboxylic acid, N-methoxycarbonyl-, undecyl ester | 1000328-90-6 | 21.49 | 1283.50 | - | 0.76±0.15 | 0.52±0.13 | 0.69±0.08 | 0.58±0.06 | 0.4±0.05 | 0.4±0.11 | 0.33±0.03 | 0.28±0 | 0.23±0.01 | 0.2±0.05 | 0.44±0.02 |
| 112 | Tetradecane, 4-methyl- | 25117-24-2 | 21.93 | 1295.65 | - | 12.9±0.6 | 11.41±0.45 | 2.44±0.13 | 2.64±0.18 | 2.68±0.58 | 1.83±0.07 | 2.11±0.21 | 2.24±0.36 | 1.66±0.15 | 0.93±0.25 | 1.18±0.19 |
| 113 | 2-Octenal, 2-butyl- | 13019-16-4 | 22.03 | 1298.73 | 1372 | 6.83±0.47 | 6.7±0.41 | 0.51±0.03 | 0.42±0.03 | 0.29±0 | 0.32±0.02 | 0.23±0.01 | 0.31±0.03 | 0.19±0.02 | 0.15±0.05 | 0.2±0.02 |
| 114 | .alfa.-Copaene | 1000360-33-0 | 22.12 | 1301.20 | 1381 | 2.42±0.14 | 2.32±0.1 | 1.14±0.05 | 1.25±0.07 | 1.19±0.2 | 1.05±0.11 | 1.15±0.1 | 1.25±0.17 | 1.01±0.12 | 0.64±0.18 | 0.7±0.04 |
| 115 | Hexanoic acid, 3-hexenyl ester, (Z)- | 31501-11-8 | 22.30 | 1306.10 | 1381 | 187.51±5.19 | 45.37±1.68 | 57.92±1.63 | 62.51±3.58 | 53.99±8.28 | 42.79±2.54 | 25.44±20 | 2.16±0.11 | 1.64±0.17 | 1.29±0.34 | 1.13±0.01 |
| 116 | cis-3-Hexenyl cis-3-hexenoate | 61444-38-0 | 22.39 | 1308.80 | 1388.8 | 45.04±2.9 | 16.49±0.92 | 6.44±0.26 | 6.02±0.47 | 4.34±0.79 | 3.32±0.27 | 2.26±0.45 | 2.04±0.13 | 1.55±0.06 | 1.26±0.44 | 1.19±0.1 |
| 117 | 5-Isoxazolecarboxylic acid, 4,5-dihydro-3,5-dimethyl-, methyl ester, (R)- | 64018-41-3 | 22.71 | 1317.63 | - | 6.79±0.35 | 8.1±2.66 | 1.12±0.19 | 2.84±3.09 | 3.85±2.34 | 4.57±3.06 | 2.86±3.04 | 1.09±0.26 | 0.91±0.09 | 0.46±0.08 | 0.56±0.06 |
| 118 | Tetradecane | 629-59-4 | 22.74 | 1318.68 | - | 65.88±2.95 | 66.28±3.66 | 15.87±0.75 | 15.64±0.88 | 15.7±3.9 | 13.6±0.77 | 14.64±1.65 | 8.38±4.58 | 11.69±1.11 | 1.08±0.32 | 4.13±3.83 |
| 119 | 2-Cyclopenten-1-one, 3-methyl-2-(2-pentenyl)-, (Z)- | 488-10-8 | 22.72 | 1318.01 | 1378 | 14.98±0.69 | 12.41±0.1 | 4.95±0.5 | 6.33±0.85 | 5.32±0.5 | 4.52±0.39 | 4.28±0.43 | 3.51±0.09 | 2.97±0.22 | 3.03±0.81 | 2.79±0.32 |
| 120 | 1H-3a,7-Methanoazulene, 2,3,4,7,8,8a-hexahydro-3,6,8,8-tetramethyl-, [3R-(3.alpha.,3a.beta.,7.beta.,8a.alpha.)]- | 469-61-4 | 23.08 | 1328.20 | 1386 | 11.71±1.1 | 9.99±0.15 | 3.78±0.42 | 4.06±0.21 | 4.43±0.79 | 3.76±0.25 | 3.99±0.58 | 4.02±0.51 | 2.94±0.33 | 2.35±0.9 | 3.56±0.39 |
| 121 | Naphthalene, 1,2-dimethyl- | 573-98-8 | 23.18 | 1331.09 | - | 1.89±0.24 | 1.68±0.06 | 0.66±0.05 | 0.63±0.12 | 0.72±0.18 | 0.61±0.06 | 0.63±0.06 | 0.43±0.06 | 0.37±0.03 | 0.35±0.04 | 0.33±0.01 |
| 122 | D-Alanine, N-(4-butylbenzoyl)-, isohexyl ester | 1000354-10-0 | 23.29 | 1334.20 | - | 4.58±0.2 | 4.16±0.1 | 1.77±0.12 | 1.89±0.14 | 1.97±0.43 | 1.64±0.12 | 1.89±0.05 | 1.69±0.18 | 1.28±0.12 | 1±0.34 | 1.42±0.08 |
| 123 | .alpha.-Ionone | 127-41-3 | 23.45 | 1338.56 | 1421 | 13.27±0.45 | 13.06±0.3 | 10.37±0.59 | 13.03±1.44 | 10.96±1.15 | 10.88±0.81 | 11.14±0.67 | 9.77±0.22 | 8.1±0.84 | 7.79±2.08 | 4.83±0.29 |
| 124 | 1,2-Benzenediol, O-(4-butylbenzoyl)-O'-(isobutoxycarbonyl)- | 1000329-73-6 | 24.03 | 1354.79 | - | 1±0.08 | 0.92±0.08 | 0.42±0.02 | 0.5±0.04 | 0.43±0.05 | 0.41±0.08 | 0.39±0.02 | 0.28±0.02 | 0.22±0.02 | 0.22±0.05 | 0.21±0.01 |
| 125 | 5,9-Undecadien-2-one, 6,10-dimethyl-, (E)- | 3796-70-1 | 24.05 | 1355.48 | 1454 | 19.2±0.54 | 17.74±0.83 | 9.99±0.53 | 11.97±1.11 | 10.65±1.34 | 10.3±0.94 | 10.25±0.65 | 8.19±0.29 | 6.66±0.61 | 6.2±1.75 | 5.45±0.18 |
| 126 | 2,6,10-Trimethyltridecane | 3891-99-4 | 24.26 | 1361.33 | 1465.1 | 22.41±3.95 | 21.23±2.42 | 4.79±0.12 | 5.84±0.71 | 5.66±1.11 | 4.89±0.35 | 5.26±0.62 | 4.89±0.97 | 3.49±0.13 | 2.27±0.79 | 2.28±1.36 |

| | | | | | | | | | | | | | | | |
|---|---|---|---|---|---|---|---|---|---|---|---|---|---|---|---|
| 127 | 1-Isopropyl-4,7-dimethyl-1,2,3,4,5,6-hexahydronaphthalene | 16729-00-3 | 24.61 | 1371.35 | 1481.3 | 2.1±0.18 | 1.87±0.16 | 0.7±0.1 | 0.8±0.06 | 0.61±0.09 | 0.7±0.15 | 0.57±0.08 | 0.42±0.04 | 0.32±0.02 | 0.26±0.09 | 0.28±0.08 |
| 128 | Acenaphthene | 83-32-9 | 24.76 | 1375.55 | 1472 | 1.14±0.22 | 1.07±0.09 | 0.42±0.03 | 0.38±0.05 | 0.51±0.12 | 0.4±0.06 | 0.45±0.01 | 0.33±0.01 | 0.27±0 | 0.27±0.08 | 0.38±0.01 |
| 129 | 3-Buten-2-one, 4-(2,6,6-trimethyl-1-cyclohexen-1-yl)- | 14901-07-6 | 24.93 | 1380.16 | 1488.4 | 123.3±4.26 | 136.52±4.35 | 127.04±8.51 | 148.73±15.77 | 126.39±10.87 | 125.72±7.16 | 124.89±7.1 | 114.76±2.49 | 99.81±8.35 | 102.8±24.23 | 64.83±5.38 |
| 130 | 3-Pyridinecarbonitrile, 1,4-dihydro-1-methyl- | 19424-15-8 | 25.10 | 1385.00 | - | 0.61±0.02 | 0.51±0.02 | 0.2±0.03 | 0.26±0.03 | 0.22±0.03 | 0.22±0.04 | 0.19±0.03 | 0.11±0.03 | 0.06±0 | 0.05±0.01 | 0.05±0 |
| 131 | Nonyl tetradecyl ether | 1000406-37-6 | 25.33 | 1391.64 | - | 7.35±6.94 | 5.94±3.43 | 0.83±0.23 | 1.01±0.27 | 0.83±0.24 | 0.56±0.22 | 1.02±0.74 | 2.28±1.05 | 0.47±0.21 | 0.4±0.08 | 0.96±0.8 |
| 132 | 2,4-Di-tert-butylphenol | 96-76-4 | 25.50 | 1396.31 | 1513 | 41.04±3.92 | 33.59±6.05 | 13.48±0.88 | 10.95±0.82 | 24.32±0.46 | 21.17±5.55 | 17.48±0.94 | 27.7±0.35 | 19.83±2.14 | 18.45±4.95 | 11.47±1.78 |
| 133 | Naphthalene, 1,2,3,4,4a,5,6,8a-octahydro-7-methyl-4-methylene-1-(1-methylethyl)-, (1.alpha.,4a.beta.,8a.alpha.)- | 39029-41-9 | 25.60 | 1399.22 | 1511 | 4±0.2 | 3.5±0.01 | 1.81±0.18 | 2.45±0.24 | 3.38±0.42 | 2.1±0.23 | 1.98±0.15 | 1.63±0.01 | 1.23±0.08 | 1.04±0.4 | 1.14±0.07 |
| 134 | 1-Decanol, 2-hexyl- | 2425-77-6 | 25.71 | 1402.15 | - | 7.01±0.26 | 3.9±0.49 | 1±0.03 | 1.07±0.19 | 1.08±0.32 | 0.81±0.15 | 0.87±0.07 | 0.81±0.07 | 0.56±0.03 | 0.35±0.15 | 0.52±0.08 |
| 135 | Naphthalene, 1,2,3,5,6,8a-hexahydro-4,7-dimethyl-1-(1-methylethyl)-, (1S-cis)- | 483-76-1 | 25.84 | 1405.95 | 1519 | 24.69±1.01 | 22.2±1.04 | 12.27±0.89 | 15.07±1.11 | 12.14±1.42 | 12.87±1.91 | 11.34±1.08 | 5.39±4.48 | 0.73±0.07 | 0.57±0.15 | 0.73±0.02 |
| 136 | Cubenene | 29837-12-5 | 26.04 | 1411.39 | 1512 | 3.37±0.27 | 3.05±0.22 | 1.49±0.22 | 1.83±0.19 | 1.56±0.15 | 1.61±0.31 | 1.4±0.14 | 1.16±0.08 | 0.87±0.03 | 0.82±0.27 | 0.94±0.04 |
| 137 | 4-Isopropyl-6-methyl-1-methylene-1,2,3,4-tetrahydronaphthalene | 50277-34-4 | 26.29 | 1418.67 | 1555 | 10.26±0.77 | 9.06±0.58 | 4.39±0.58 | 5.22±0.46 | 4.45±0.58 | 4.43±0.86 | 3.87±0.61 | 2.79±0.2 | 2.12±0.07 | 1.9±0.6 | 2.27±0.13 |
| 138 | n-Nonylcyclohexane | 2883-2-5 | 26.40 | 1421.72 | 1556 | 13.19±0.24 | 9.18±0.94 | 2.22±0.09 | 2.33±0.29 | 2.34±0.69 | 1.89±0.24 | 1.93±0.23 | 1.33±0.48 | 1.24±0.15 | 0.83±0.28 | 1.01±0.08 |
| 139 | Oxazole | 288-42-6 | 26.40 | 1421.65 | - | 5.96±0.5 | 3.81±1.18 | 1.28±0.28 | 1.17±0.14 | 1.21±0.37 | 1.18±0.39 | 1.25±0.3 | 1.05±0.01 | 0.8±0.16 | 0.26±0.1 | 0.69±0.14 |
| 140 | Hexadecane, 2-methyl- | 1560-92-5 | 26.73 | 1430.93 | - | 21.9±2.02 | 15.22±1.67 | 4.4±0.18 | 4.38±0.49 | 4.67±0.94 | 3.9±0.68 | 4.04±0.67 | 3.02±0.16 | 2.63±0.04 | 1.94±0.76 | 1.59±0.61 |
| 141 | 1,6,10-Dodecatrien-3-ol, 3,7,11-trimethyl- | 7212-44-4 | 26.73 | 1430.99 | 1565.6 | 11.72±0.67 | 6.12±0.36 | 1.75±0.18 | 1.77±0.17 | 1.82±0.23 | 1.47±0.19 | 1.35±0.21 | 1.09±0.08 | 0.92±0.06 | 0.75±0.2 | 0.63±0.04 |
| 142 | Pentadecane, 3-methyl- | 2882-96-4 | 26.86 | 1434.54 | 1571 | 15.91±0.58 | 11.53±0.58 | 2.65±0.09 | 2.74±0.31 | 2.9±0.84 | 2.23±0.32 | 2.53±0.33 | 2.06±0.05 | 1.47±0.13 | 0.99±0.36 | 1.21±0.19 |
| 143 | 3-Hexen-1-ol, benzoate, (Z)- | 25152-85-6 | 26.90 | 1435.85 | 1568 | 6.3±0.56 | 2.16±0.07 | 0.98±0.15 | 1.1±0.15 | 1.24±0.2 | 1±0.08 | 0.88±0.05 | 0.77±0.01 | 0.65±0.04 | 0.65±0.15 | 0.58±0.06 |
| 144 | Pentadecane, 1-methoxy-13-methyl- | 56196-09-9 | 27.21 | 1444.52 | - | 20.93±1.31 | 16.21±1.24 | 3.39±0.12 | 3.68±0.46 | 3.38±0.99 | 3.15±0.56 | 3.47±0.64 | 3±0.06 | 2.11±0.27 | 1.4±0.55 | 1.69±0.21 |
| 145 | 1-Hexadecanol | 36653-82-4 | 27.36 | 1448.68 | - | 4.26±0.54 | 3.38±0.47 | 0.74±0.11 | 0.7±0.08 | 0.61±0.24 | 0.67±0.25 | 0.63±0.08 | 0.57±0.08 | 0.4±0.07 | 0.41±0.16 | 0.39±0.1 |
| 146 | Dodecanoic acid, ethyl ester | 106-33-2 | 27.43 | 1450.58 | 1597 | 5.36±0.47 | 6.16±0.58 | 0.77±0.11 | 1.04±0.13 | 0.84±0.02 | 0.82±0.06 | 0.78±0.06 | 0.68±0.02 | 0.55±0.01 | 0.59±0.06 | 0.61±0.04 |
| 147 | Hexadecane | 544-76-3 | 27.55 | 1454.00 | - | 31.48±1.38 | 26.48±2.84 | 6.78±0.34 | 7.38±0.65 | 7.25±1.54 | 6.65±1.42 | 6.56±0.39 | 5.87±0.22 | 4.53±0.36 | 0.86±0.39 | 1.39±0.28 |
| 148 | 5,5-Diethyltridecane | 1000360-41-3 | 27.60 | 1455.58 | 1605 | 7.39±0.11 | 4.26±0.53 | 0.82±0.02 | 1.09±0.17 | 0.94±0.31 | 0.71±0.11 | 0.88±0.14 | 1.16±0.06 | 0.71±0.09 | 0.36±0.15 | 0.54±0.08 |
| 149 | Cedrol | 77-53-2 | 27.68 | 1457.72 | 1605.8 | 4.02±0.5 | 3.43±1 | 1.54±0.05 | 1.63±0.14 | 1.93±0.58 | 1.63±0.29 | 2.13±0.18 | 1.6±0.1 | 0.93±0.01 | 1.01±0.37 | 1.38±0.23 |
| 150 | Di-epi-1,10-cubenol | 73365-77-2 | 28.27 | 1474.27 | 1614 | 4.03±0.23 | 3.36±0.11 | 1.74±0.25 | 2.02±0.23 | 1.73±0.19 | 1.69±0.25 | 1.41±0.22 | 1.12±0.04 | 0.93±0.02 | 0.88±0.23 | 0.89±0.05 |
| 151 | 1,2-Benzenediol, O-acetoxyacetyl-O'-(4-butylbenzoyl)- | 1000330-49-5 | 28.55 | 1482.06 | - | 1.84±0.09 | 1.48±0.07 | 0.78±0.1 | 1.07±0.12 | 0.96±0.07 | 0.8±0.1 | 0.79±0.13 | 0.58±0.06 | 0.48±0.01 | 0.45±0.11 | 0.44±0.04 |

| | | | | | | | | | | | | | | | | |
|---|---|---|---|---|---|---|---|---|---|---|---|---|---|---|---|---|
| 152 | 3,3-Diethyltridecane | 1000360-41-2 | 28.70 | 1486.43 | 1653 | 4.54±0.1 | 2.54±0.4 | 0.47±0.06 | 0.65±0.1 | 0.59±0.2 | 0.45±0.08 | 0.57±0.1 | 0.65±0 | 0.39±0.04 | 0.23±0.1 | 0.31±0.02 |
| 153 | .alpha.-Cadinol | 481-34-5 | 28.85 | 1490.73 | 1653 | 1.97±0.14 | 1.35±0.04 | 0.93±0.16 | 1.43±0.21 | 1.32±0.07 | 1.06±0.16 | 1.11±0.21 | 0.74±0.09 | 0.63±0.01 | 0.56±0.15 | 0.55±0.05 |
| 154 | 1,1'-Biphenyl, 2,2',5,5'-tetramethyl- | 3075-84-1 | 29.37 | 1505.29 | 1656.5 | 3.09±0.1 | 2.52±0.52 | 0.88±0.11 | 0.97±0.14 | 1.29±0.33 | 1.04±0.19 | 1.48±0.05 | 0.91±0.03 | 0.55±0 | 0.56±0.19 | 0.87±0.09 |
| 155 | 7-Methyl-octadecane | 1000192-63-3 | 29.90 | 1520.09 | - | 15.35±0.17 | 10.12±1.38 | 1.94±0.05 | 2.03±0.35 | 2.94±0.81 | 2.11±0.58 | 2.73±0.03 | 1.55±0.15 | 1.28±0.01 | 0.94±0.38 | 1.3±0.08 |
| 156 | 2-Hexyl-5-pentylpyrrolidine | 73427-38-0 | 30.27 | 1530.73 | - | 1.23±0.08 | 0.77±0.14 | 0.17±0.01 | 0.15±0.02 | 0.21±0.09 | 0.15±0.03 | 0.2±0.02 | 0.12±0.01 | 0.07±0.01 | 0.06±0.02 | 0.06±0.01 |
| 157 | Hexadecane, 1-iodo- | 544-77-4 | 30.74 | 1543.79 | - | 8±1.02 | 5.37±0.1 | 0.9±0.46 | 1.29±0.17 | 1.43±0.4 | 0.72±0.37 | 0.99±0.47 | 1.11±0.1 | 0.64±0.01 | 0.44±0.17 | 0.43±0.04 |
| 158 | Cyclohexane, undecyl- | 54105-66-7 | 31.04 | 1552.31 | 1760 | 8.25±0.49 | 4.98±0.67 | 1.53±0.06 | 1.41±0.15 | 1.68±0.57 | 1.38±0.26 | 1.62±0.21 | 1.08±0.08 | 0.71±0.01 | 0.59±0.18 | 0.55±0.04 |
| 159 | Heptadecane, 3-methyl- | 6418-44-6 | 31.28 | 1559.18 | 1771 | 4.6±0.46 | 2.88±0.43 | 0.75±0.06 | 0.69±0.08 | 0.8±0.27 | 0.64±0.13 | 0.69±0.11 | 0.46±0.04 | 0.31±0.01 | 0.27±0.09 | 0.24±0.01 |
| 160 | 2,3,4-Trimethyl-1-pentanol | 6570-88-3 | 31.62 | 1568.60 | - | 4.48±0.06 | 2.55±0.46 | 0.64±0.06 | 0.6±0.1 | 0.72±0.27 | 0.57±0.17 | 0.59±0.11 | 0.48±0.04 | 0.28±0.01 | 0.22±0.07 | 0.22±0.01 |
| 161 | Cyclohexane, 1-isopropyl-1-methyl- | 16580-26-0 | 31.75 | 1572.28 | - | 3.66±0.41 | 2.21±0.44 | 0.61±0.08 | 0.59±0.05 | 0.75±0.24 | 0.59±0.15 | 0.68±0.08 | 0.48±0.02 | 0.27±0.02 | 0.26±0.08 | 0.23±0.02 |
| 162 | Tetradecanoic acid, ethyl ester | 124-06-1 | 31.79 | 1573.30 | 1793 | 4.54±1.03 | 4.65±0.48 | 0.49±0.12 | 0.55±0.08 | 0.47±0.05 | 0.39±0.04 | 0.36±0.06 | 0.29±0.02 | 0.19±0 | 0.2±0.07 | 0.23±0.02 |
| 163 | Octadecane | 593-45-3 | 31.89 | 1576.19 | - | 4.5±0.46 | 3.01±0.03 | 0.81±0.18 | 0.96±0.08 | 1.09±0.26 | 0.92±0.19 | 0.81±0.27 | 0.42±0.04 | 0.35±0 | 0.37±0.1 | 0.28±0.01 |
| 164 | 5,5-Diethylpentadecane | 1000360-41-0 | 31.97 | 1578.53 | 1805 | 2.54±0.09 | 1.1±0.22 | 0.26±0.02 | 0.31±0.05 | 0.28±0.11 | 0.22±0.05 | 0.29±0.05 | 0.34±0.02 | 0.18±0 | 0.11±0.03 | 0.13±0.01 |
| 165 | Hexadecane, 2,6,10,14-tetramethyl- | 638-36-8 | 32.09 | 1581.77 | 1811 | 9.5±0.66 | 5.94±1.31 | 1.27±0.11 | 1.46±0.12 | 2.06±0.57 | 1.58±0.49 | 1.89±0.48 | 1.12±0.13 | 0.81±0.03 | 0.66±0.21 | 0.88±0.12 |
| 166 | Neophytadiene | 504-96-1 | 32.70 | 1599.19 | - | 8.91±3.22 | 6.16±1.03 | 1.5±0.46 | 2.15±0.43 | 3.64±0.79 | 2.7±0.76 | 2.37±0.82 | 1.05±0.15 | 0.71±0.03 | 0.7±0.25 | 0.57±0.06 |
| 167 | Phthalic acid, hept-4-yl isobutyl ester | 1000356-78-3 | 33.32 | 1616.56 | - | 19.98±2.39 | 13.67±1.91 | 6.37±0.99 | 6.66±1.02 | 5.67±1.53 | 4.43±1.18 | 5.55±1.14 | 4.43±0.14 | 2.66±0.85 | 2.45±0.24 | 2.06±0.25 |
| 168 | Hexadecanoic acid, methyl ester | 112-39-0 | 34.44 | 1647.99 | 1920 | 5.34±1.1 | 2.58±0.15 | 0.94±0.17 | 1±0.15 | 1.04±0.17 | 0.82±0.14 | 0.78±0.14 | 0.48±0.04 | 0.41±0.01 | 0.37±0.1 | 0.83±0.02 |
| 169 | Phthalic acid, butyl hept-4-yl ester | 1000356-78-4 | 35.17 | 1668.48 | - | 0.83±0.06 | 0.56±0.07 | 0.95±1.13 | 0.45±0.12 | 0.36±0.12 | 0.3±0.05 | 0.27±0.06 | 0.18±0 | 0.1±0.01 | 0.1±0.02 | 0.1±0.01 |
| 170 | Hexadecanoic acid, ethyl ester | 628-97-7 | 35.76 | 1685.13 | 1993 | 18.62±2.73 | 14.72±1.06 | 4.06±0.72 | 4.35±0.61 | 4.7±0.66 | 3.6±0.52 | 2.92±0.56 | 2.07±0.18 | 1.38±0.07 | 1.35±0.38 | 1.7±0.16 |
| 171 | Phytol | 150-86-7 | 37.95 | 1877.40 | 2122 | 18.36±3.64 | 12.48±0.94 | 2.62±0.98 | 4.82±0.49 | 7.91±0.54 | 5.55±1.81 | 4.6±2.66 | 1.82±0.86 | 0.95±0.14 | 0.85±0.32 | 0.77±0.13 |
| 172 | Linoleic acid ethyl ester | 544-35-4 | 38.82 | 1964.04 | 2193 | 0.71±0.13 | 0.46±0.06 | 0.2±0.06 | 0.28±0.05 | 0.34±0 | 0.28±0.04 | 0.21±0.07 | 0.12±0.03 | 0.07±0.01 | 0.07±0.03 | 0.07±0 |
| 173 | Ethyl 9,12,15-octadecatrienoate | 1000336-77-4 | 38.94 | 1975.80 | 2101 | 3.97±0.69 | 3.1±0.53 | 0.97±0.26 | 1.36±0.17 | 1.59±0.01 | 1.32±0.2 | 1.02±0.34 | 0.67±0.16 | 0.46±0.02 | 0.44±0.14 | 0.52±0.06 |

$RI^A$: the linear retention indices calculated from a series of n-alkanes (C7-C40).
$RI^B$: retention indices referred to the literature value with same or equivalent chromatographic column shown on NIST Chemistry WebBook (http://webbook.nist.gov/chemistry/).

**Table S2**. List all of the volatile compounds in round green tea by different second-drying temperature.

| NO | Name | CAS | RT | RI[A] | RI[B] | BHL70 | BHL90 | BHL110 |
|---|---|---|---|---|---|---|---|---|
| 1 | Dimethyl ether | 115-10-6 | 2.15 | 738.693 | - | 8.59±1.05 | 8.12±0.64 | 7.33±1.87 |
| 2 | Butanal, 3-methyl- | 590-86-3 | 2.17 | 743.0526 | 682 | 3.8±1.03 | 3.34±0.59 | 3.69±0.32 |
| 3 | Butanal, 2-methyl- | 96-17-3 | 2.16 | 739.0089 | 678 | 5.29±1.68 | 4.63±0.85 | 6.42±0.79 |
| 4 | 1-Penten-3-ol | 616-25-1 | 2.30 | 751.7714 | 685 | 14.22±2.86 | 11.46±1.71 | 8.7±1.13 |
| 5 | 1-Penten-3-one | 1629-58-9 | 2.61 | 753.3194 | 678 | 3.62±0.75 | 3.48±0.91 | 2.29±0.08 |
| 6 | Pentanal | 110-62-3 | 2.67 | 757.0788 | 701 | 6.88±2.01 | 5.41±1.58 | 2.59±0.07 |
| 7 | Furan, 2-ethyl- | 3208-16-0 | 2.80 | 758.9111 | 702 | 1.96±0.67 | 1.37±0.31 | 0.98±0.12 |
| 8 | 3-Penten-2-one | 625-33-2 | 2.87 | 778.9078 | 744 | 1.02±0.2 | 1.02±0.1 | 0.98±0.02 |
| 9 | Cyclobutaneethanol, beta-methylene- | 116203-80-6 | 3.41 | 787.5318 | - | 0.99±0.23 | 0±0 | 0±0 |
| 10 | Toluene | 108-88-3 | 3.58 | 793.8816 | 773 | 3.1±0.85 | 1.41±0.42 | 2.35±0.15 |
| 11 | 1-Pentanol | 71-41-0 | 3.88 | 795.1452 | 779 | 11.52±2.64 | 10.84±2.23 | 5.29±0.28 |
| 12 | 2-Penten-1-ol, (Z)- | 1576-95-0 | 3.88 | 797.5144 | 769 | 7.93±1.37 | 6.71±0.85 | 4.91±0.32 |
| 13 | Furan, 2-methoxy- | 25414-22-6 | 4.11 | 816.1211 | - | 6.36±1.67 | 10.75±1.29 | 8.45±0.85 |
| 14 | Hexanal | 66-25-1 | 4.15 | 816.816 | 800 | 4.82±1.24 | 10.71±2.47 | 5.64±0.64 |
| 15 | 1H-Pyrrole, 3-ethyl- | 1551-16-2 | 4.22 | 827.8411 | - | 1.19±0.25 | 2.04±0.16 | 9.84±1.34 |
| 16 | 2-Hexenal, (E)- | 6728-26-3 | 4.24 | 859.5583 | 854 | 1.01±0.22 | 0.87±0.2 | 0.5±0.03 |
| 17 | 3-Hexen-1-ol, (E)- | 928-97-2 | 4.89 | 862.1171 | 844 | 7.95±1.3 | 6.68±0.72 | 2.88±0.37 |
| 18 | Ethylbenzene | 100-41-4 | 4.90 | 872.1315 | 857 | 1.72±0.43 | 0±0 | 0±0 |
| 19 | 1-Hexanol | 111-27-3 | 4.92 | 873.9084 | 867 | 7.1±1.33 | 1.66±0.18 | 2.89±0.28 |
| 20 | Ethanone, 1-(1-cyclohexen-1-yl)- | 932-66-1 | 4.92 | 887.9898 | - | 0.36±0.09 | 0.43±0.04 | 0.39±0.13 |
| 21 | Styrene | 100-42-5 | 5.31 | 890.7382 | 890 | 3.57±1.25 | 0±0 | 2.16±0.15 |
| 22 | 2-Heptanone | 110-43-0 | 5.71 | 892.5071 | 895 | 2.15±0.61 | 0±0 | 1.34±0.15 |
| 23 | 4-Heptenal, (E)- | 929-22-6 | 6.44 | 899.8678 | 905 | 1.34±0.31 | 0±0 | 0.57±0.06 |
| 24 | Heptanal | 111-71-7 | 6.53 | 901.4787 | 901 | 17.82±3.73 | 11.93±1.86 | 2.11±0.16 |
| 25 | Oxime-, methoxy-phenyl-_ | 1000222-86-6 | 6.68 | 910.1351 | - | 25.42±3.58 | 23.02±4.16 | 13.25±1.42 |
| 26 | 1-Butanol, 3-methyl-, nitrate | 543-87-3 | 6.89 | 923.9402 | - | 1.73±0.59 | 1.15±0.4 | 0±0 |
| 27 | Benzene, 1-methoxy-4-methyl- | 104-93-8 | 6.95 | 931.68 | - | 1.17±0.34 | 1.02±0.05 | 1.14±0.11 |
| 28 | 2-Heptenal, (E)- | 18829-55-5 | 7.21 | 950.9435 | 956 | 8.29±2.67 | 9.78±2.84 | 1.8±0.39 |
| 29 | Benzaldehyde | 100-52-7 | 7.22 | 953.1889 | 961 | 6.81±0.86 | 3.13±0.27 | 3.71±0.09 |
| 30 | 1-Heptanol | 111-70-6 | 7.45 | 964.567 | 969 | 2.66±0.49 | 2.33±0.22 | 1.64±0.17 |
| 31 | 2-Hexene, 3,5,5-trimethyl- | 26456-76-8 | 7.54 | 967.3559 | - | 2.85±1.01 | 9.41±1.79 | 5.2±0.76 |
| 32 | 1-Octen-3-ol | 3391-86-4 | 7.55 | 972.8435 | 986 | 37.29±10.85 | 36.03±7.06 | 21.57±2.98 |
| 33 | Pentanoic acid, 2-methyl-, anhydride | 63169-61-9 | 7.61 | 977.835 | - | 29.52±5.16 | 25.41±3.4 | 18.9±1.11 |
| 34 | 5-Hepten-2-one, 6-methyl- | 110-93-0 | 7.87 | 979.8887 | 988 | 7.74±1.88 | 6.74±0.99 | 4.92±0.25 |
| 35 | .beta.-Myrcene | 123-35-3 | 7.87 | 983.3215 | 981 | 10.07±1.75 | 9.06±0.82 | 12.99±1.1 |
| 36 | Furan, 2-pentyl- | 3777-69-3 | 7.93 | 983.4586 | 996 | 23.18±6.87 | 16.45±1.91 | 14.98±1.49 |
| 37 | Hexanoic acid, ethyl ester | 123-66-0 | 8.20 | 991.7675 | 998 | 3.54±0.67 | 18.62±15.13 | 1.36±0.22 |
| 38 | cis-2-(2-Pentenyl)furan | 70424-13-4 | 8.24 | 992.2099 | - | 0.9±0.22 | 0±0 | 0±0 |

| | | | | | | | | |
|---|---|---|---|---|---|---|---|---|
| 39 | Octanal | 124-13-0 | 8.24 | 993.6631 | 1004 | 4.11±1.04 | 2.77±0.38 | 0±0 |
| 40 | 2,4-Heptadienal, (E,E)- | 4313-03-5 | 8.35 | 999.9474 | 1008 | 7.28±1.56 | 6.36±1.27 | 1.09±0.08 |
| 41 | o-Cymene | 527-84-4 | 8.73 | 1012.177 | 1021 | 2.24±0.41 | 2.59±0.53 | 3.73±0.3 |
| 42 | D-Limonene | 5989-27-5 | 9.00 | 1015.678 | 1035 | 3.35±0.55 | 1.78±0.4 | 7.11±0.76 |
| 43 | 1-Hexanol, 2-ethyl- | 104-76-7 | 9.68 | 1017.295 | 1029 | 7.85±1.25 | 8.85±1.09 | 3.7±0.07 |
| 44 | 5-Ethylcyclopent-1-enecarboxaldehyde | 36431-60-4 | 9.76 | 1018.338 | 1026 | 0.82±0.13 | 0.76±0.1 | 0.62±0.02 |
| 45 | Cyclohexanone, 2,2,6-trimethyl- | 2408-37-9 | 9.93 | 1020.802 | 1022.9 | 6.49±1.84 | 4.72±0.91 | 3.28±0.3 |
| 46 | Benzyl alcohol | 100-51-6 | 10.17 | 1021.592 | 1033 | 16.81±1.72 | 17.36±1.77 | 16.84±0.51 |
| 47 | Bicyclo[3.1.1]hept-2-ene, 3,6,6-trimethyl- | 4889-83-2 | 10.27 | 1025.572 | - | 1.6±0.31 | 1.62±0.23 | 3.35±0.39 |
| 48 | Benzeneacetaldehyde | 122-78-1 | 10.43 | 1029.3 | 1043 | 14.21±1.74 | 12.66±2.67 | 16.02±0.53 |
| 49 | Phenol, 4-amino-3-methyl- | 2835-99-6 | 10.46 | 1034.513 | - | 2.92±0.58 | 17.94±0.59 | 56.94±2.59 |
| 50 | 3-Carene | 13466-78-9 | 10.57 | 1034.513 | 1005 | 2.92±0.58 | 3.3±0.27 | 0±0 |
| 51 | 1-Undecene, 9-methyl- | 74630-41-4 | 10.63 | 1042.66 | - | 4.84±1.22 | 0±0 | 0±0 |
| 52 | Acetophenone | 98-86-2 | 10.64 | 1049.172 | 1065 | 3.09±0.41 | 2.99±0.26 | 2.34±0.06 |
| 53 | Cyclooctyl alcohol | 696-71-9 | 10.71 | 1052.142 | - | 6.17±1.76 | 7.17±1.15 | 5.18±0.97 |
| 54 | 1-Octanol | 111-87-5 | 10.83 | 1054.827 | 1068 | 11.74±1.97 | 9.92±0.83 | 0±0 |
| 55 | Ethyl 2-(5-methyl-5-vinyltetrahydrofuran-2-yl)propan-2-yl carbonate | 1000373-80-3 | 10.84 | 1069.47 | - | 4.68±0.69 | 8.59±0.43 | 8.36±0.68 |
| 56 | 3,5-Octadien-2-one | 38284-27-4 | 11.11 | 1073.814 | 1093 | 2.03±0.31 | 1.81±0.02 | 2.15±0.14 |
| 57 | Linalool | 78-70-6 | 11.13 | 1080.196 | 1080 | 23.79±3.93 | 22.73±0.9 | 22.34±0.83 |
| 58 | Nonanal | 124-19-6 | 11.15 | 1084.145 | 1102 | 29.97±4.34 | 29.62±1.38 | 0±0 |
| 59 | Phenylethyl Alcohol | 60-12-8 | 11.20 | 1090.464 | 1116 | 15.1±1.63 | 9.73±0.91 | 26.61±1.28 |
| 60 | Ethanone, 1-(4-methylphenyl)- | 122-00-9 | 11.42 | 1105.345 | 1168 | 0.37±0.07 | 0±0 | 0±0 |
| 61 | Benzene, 1-isocyano-3-methyl- | 20600-54-8 | 11.60 | 1112.548 | - | 2.46±0.35 | 0±0 | 0±0 |
| 62 | Decane, 2,3,5-trimethyl- | 62238-11-3 | 11.86 | 1117.509 | - | 0.92±0.23 | 0±0 | 0±0 |
| 63 | (R,S)-5-Ethyl-6-methyl-3E-hepten-2-one | 57283-79-1 | 11.98 | 1119.436 | 1143.9 | 1.97±0.31 | 1.65±0.14 | 1.09±0.06 |
| 64 | 2-Oxo-4-phenyl-6-(4-chlorophenyl)-1,2-dihydropyrimidine | 24030-13-5 | 12.04 | 1129.389 | - | 5.34±0.94 | 14.86±6.17 | 70.66±25.62 |
| 65 | 1-Nonanol | 143-08-8 | 12.08 | 1140.289 | 1186 | 2.3±0.37 | 2.26±0.07 | 2.08±0.05 |
| 66 | (3R,6S)-2,2,6-Trimethyl-6-vinyltetrahydro-2H-pyran-3-ol | 39028-58-5 | 12.17 | 1142.595 | 1183 | 11.1±1.32 | 11.27±0.83 | 12.03±0.38 |
| 67 | Benzaldehyde, 3-ethyl- | 34246-54-3 | 12.19 | 1150.683 | 1168 | 0.28±0.06 | 0.32±0.01 | 0±0 |
| 68 | cis-3-Hexenyl iso-butyrate | 41519-23-7 | 12.33 | 1153.179 | - | 2±0.3 | 0±0 | 0±0 |
| 69 | .alpha.-Terpineol | 98-55-5 | 12.36 | 1155.991 | 1190 | 1.37±0.16 | 0±0 | 0±0 |
| 70 | Methyl salicylate | 119-36-8 | 12.47 | 1158.941 | 1187 | 16.03±1.95 | 16.54±0.34 | 18.62±0.76 |
| 71 | Octanoic acid, ethyl ester | 106-32-1 | 12.65 | 1181.045 | 1199 | 38.92±8.06 | 29.2±2.05 | 0±0 |
| 72 | 1,3-Cyclohexadiene-1-carboxaldehyde, 2,6,6-trimethyl- | 116-26-7 | 12.65 | 1163.479 | 1178 | 2.12±0.47 | 1.56±0.01 | 2.64±0.17 |
| 73 | Dodecane | 112-40-3 | 12.94 | 1163.637 | - | 5.25±0.96 | 6.25±0.33 | 5.08±0.69 |
| 74 | Decanal | 112-31-2 | 12.94 | 1168.471 | 1200 | 1.9±0.28 | 2.79±0.23 | 1.64±0.03 |
| 75 | Formamide, N-phenyl- | 103-70-8 | 12.95 | 1175.8 | - | 0.33±0.07 | 0.34±0.05 | 0.44±0.03 |

| | | | | | | | | |
|---|---|---|---|---|---|---|---|---|
| 76 | 1-Cyclohexene-1-carboxaldehyde, 2,6,6-trimethyl- | 432-25-7 | 12.96 | 1180.54 | 1214 | 10.97±1.72 | 9.82±0.75 | 8.64±0.44 |
| 77 | 2,6-Octadien-1-ol, 3,7-dimethyl-, (Z)- | 106-25-2 | 13.04 | 1208.091 | 1229 | 65.7±7.9 | 0±0 | 67.55±2.86 |
| 78 | n-Valeric acid cis-3-hexenyl ester | 35852-46-1 | 13.05 | 1190.271 | 1235.8 | 5.11±0.84 | 4.04±0.14 | 3.49±0.24 |
| 79 | trans-2-Hexenyl isovalerate | 68698-59-9 | 13.05 | 1195.105 | 1244.9 | 0.82±0.17 | 0±0 | 0±0 |
| 80 | 6-Undecanone | 927-49-1 | 13.12 | 1198.802 | 1274 | 18.39±3.07 | 17.93±2.25 | 0±0 |
| 81 | Geraniol | 106-24-1 | 13.17 | 1208.091 | 1267 | 65.81±7.88 | 61.66±3.29 | 67.71±2.8 |
| 82 | 1-Cyclohexene-1-acetaldehyde, 2,6,6-trimethyl- | 472-66-2 | 13.28 | 1210.082 | 1254 | 1.9±0.32 | 1.64±0.17 | 2.55±0.77 |
| 83 | Dodecane, 4,6-dimethyl- | 61141-72-8 | 13.37 | 1227.397 | - | 2.27±0.61 | 0.83±1.04 | 0±0 |
| 84 | 1-Decanol | 112-30-1 | 13.39 | 1236.086 | 1272 | 0.58±0.17 | 0±0 | 0±0 |
| 85 | Indole | 120-72-9 | 13.62 | 1237.129 | 1294 | 0.87±0.1 | 11.98±1.36 | 5.48±0.23 |
| 86 | Tridecane | 629-50-5 | 13.80 | 1242.532 | - | 2.12±0.33 | 4.74±1.05 | 2.81±0.12 |
| 87 | Nonane, 5-butyl- | 17312-63-9 | 13.89 | 1245.849 | - | 0.98±0.16 | 0±0 | 0±0 |
| 88 | Naphthalene, 2-methyl- | 91-57-6 | 13.92 | 1249.609 | 1315.3 | 0.66±0.07 | 0.92±0 | 0.71±0.02 |
| 89 | Heptadecane, 7-methyl- | 20959-33-5 | 13.94 | 1269.642 | - | 1.33±0.16 | 0±0 | 0±0 |
| 90 | Methyl anthranilate | 134-20-3 | 14.05 | 1273.845 | 1338 | 0.42±0.05 | 0.46±0.05 | 0.78±0.06 |
| 91 | Hexane, 2,2,5,5-tetramethyl- | 1071-81-4 | 14.27 | 1282.438 | - | 0.89±0.15 | 0±0 | 0±0 |
| 92 | .alpha.-Cubebene | 17699-14-8 | 14.41 | 1281.175 | 1354 | 2.87±0.34 | 2.87±0.14 | 3.6±0.25 |
| 93 | Naphthalene, 1,2-dihydro-4,5,7-trimethyl- | 53156-11-9 | 14.46 | 1283.197 | - | 0.59±0.07 | 0±0 | 0±0 |
| 94 | Tridecane, 3-methyl- | 6418-41-3 | 14.51 | 1295.241 | 1371 | 5.64±0.6 | 0±0 | 4.93±0.65 |
| 95 | Cubenene | 29837-12-5 | 14.53 | 1300.891 | 1552 | 0.99±0.1 | 0±0 | 0±0 |
| 96 | Hexanoic acid, 3-hexenyl ester, (Z)- | 31501-11-8 | 14.64 | 1303.925 | 1381 | 22.65±1.97 | 20.06±0.61 | 21.7±0.87 |
| 97 | Hexanoic acid, hexyl ester | 6378-65-0 | 15.14 | 1307.384 | 1385 | 2.57±0.23 | 2.59±0.05 | 2.66±0.14 |
| 98 | Hexanoic acid, 2-hexenyl ester, (E)- | 53398-86-0 | 15.16 | 1309.842 | 1391 | 3.75±0.41 | 0±0 | 3.74±0.37 |
| 99 | 1-Dodecanol | 112-53-8 | 15.17 | 1311.161 | 1469 | 1.11±0.11 | 0±0 | 0±0 |
| 100 | 2-Cyclopenten-1-one, 3-methyl-2-(2-pentenyl)-, (Z)- | 488-10-8 | 15.18 | 1317.322 | 1396 | 2.86±0.38 | 2.75±0.33 | 3.33±0.2 |
| 101 | Tetradecane | 629-59-4 | 15.34 | 1317.544 | - | 9.28±1.13 | 7.84±0.68 | 8.04±1.13 |
| 102 | 1H-3a,7-Methanoazulene, 2,3,4,7,8,8a-hexahydro-3,6,8,8-tetramethyl-, [3R-(3.alpha.,3a.beta.,7.beta.,8a.alpha.)]- | 469-61-4 | 15.42 | 1327.813 | 1408 | 3.34±0.28 | 3.56±0.39 | 3.57±0.22 |
| 103 | D-Alanine, N-(4-butylbenzoyl)-, isohexyl ester | 1000354-10-0 | 15.42 | 1333.817 | - | 1.41±0.11 | 1.42±0.08 | 0±0 |
| 104 | .alpha.-Ionone | 127-41-3 | 15.43 | 1338.115 | 1426 | 5.02±0.54 | 4.83±0.29 | 5.25±0.32 |
| 105 | Pentafluoropropionic acid, tetradecyl ester | 6222-06-6 | 15.52 | 1351.07 | - | 1.78±0.21 | 2.15±0.38 | 1.55±0.06 |
| 106 | 5,9-Undecadien-2-one, 6,10-dimethyl-, (E)- | 3796-70-1 | 15.58 | 1355.019 | 1454 | 5.3±0.62 | 5.45±0.18 | 6.3±0.3 |
| 107 | 2,6,10-Trimethyltridecane | 3891-99-4 | 15.60 | 1360.896 | 1465.1 | 3.06±0.23 | 3.06±0.42 | 3.14±0.27 |
| 108 | Cyclopentane, pentyl- | 3741-00-2 | 15.60 | 1369.049 | 1033 | 0.39±0.06 | 0±0 | 0±0 |
| 109 | Acenaphthene | 83-32-9 | 15.67 | 1375.179 | 1472 | 0.29±0.05 | 0.39±0.01 | 0.54±0.04 |
| 110 | trans-.beta.-Ionone | 79-77-6 | 16.02 | 1379.318 | 1469 | 67.74±7.94 | 64.82±5.39 | 71.24±4.14 |
| 111 | n-Pentadecanol | 629-76-5 | 16.23 | 1381.625 | 1772 | 0.91±0.11 | 0±0 | 0±0 |
| 112 | Pentadecane | 629-62-9 | 16.38 | 1386.919 | - | 2.89±0.36 | 3.25±0.46 | 3.39±0.44 |
| 113 | 2,4-Di-tert-butylphenol | 96-76-4 | 16.41 | 1395.845 | 1513 | 16.35±2.1 | 11.47±1.77 | 0±0 |

| | | | | | | | | |
|---|---|---|---|---|---|---|---|---|
| 114 | Naphthalene, 1,2,3,4,4a,5,6,8a-octahydro-7-methyl-4-methylene-1-(1-methylethyl)-, (1.alpha.,4a.beta.,8a.alpha.)- | 39029-41-9 | 16.48 | 1398.815 | 1511 | 0.99±0.08 | 1.16±0.05 | 1.45±0.03 |
| 115 | Naphthalene, 1,2,3,5,6,8a-hexahydro-4,7-dimethyl-1-(1-methylethyl)-, (1S-cis)- | 483-76-1 | 16.49 | 1404.818 | 1519 | 10.29±0.81 | 11.13±0.51 | 9.04±0.63 |
| 116 | 2(4H)-Benzofuranone, 5,6,7,7a-tetrahydro-4,4,7a-trimethyl-, (R)- | 17092-92-1 | 16.62 | 1408.768 | 1525 | 2.73±0.27 | 2.92±0.5 | 3.96±0.53 |
| 117 | 4-Isopropyl-6-methyl-1-methylene-1,2,3,4-tetrahydronaphthalene | 50277-34-4 | 16.69 | 1418.247 | 1561 | 1.91±0.15 | 2.27±0.14 | 0±0 |
| 118 | n-Nonylcyclohexane | 2883-02-5 | 16.70 | 1421.06 | 1556 | 2.31±0.25 | 2.36±0.49 | 2.48±0.16 |
| 119 | Nerolidol 2 | 1000285-43-6 | 16.75 | 1430.508 | - | 2.77±0.18 | 1.75±0.17 | 0±0 |
| 120 | Pentadecane, 3-methyl- | 2882-96-4 | 16.78 | 1434.047 | 1570 | 4.61±0.5 | 4.73±0.64 | 6.41±0.38 |
| 121 | 3-Hexen-1-ol, benzoate, (Z)- | 25152-85-6 | 16.86 | 1435.405 | 1568 | 0.55±0.05 | 0.59±0.05 | 0.9±0.08 |
| 122 | Glutaric acid, butyl cis-hex-3-enyl ester | 1000359-96-4 | 16.87 | 1439.766 | - | 4.21±0.54 | 0±0 | 0±0 |
| 123 | Ethylene diacrylate | 2274-11-5 | 16.88 | 1448.298 | - | 0.36±0.09 | 0±0 | 0±0 |
| 124 | Hexadecane | 544-76-3 | 16.97 | 1453.164 | - | 4.55±0.37 | 4.17±0.27 | 6.17±0.21 |
| 125 | 5,5-Diethyltridecane | 1000360-41-3 | 16.97 | 1455.186 | - | 0.45±0.06 | 0.54±0.08 | 0.58±0.03 |
| 126 | Cedrol | 77-53-2 | 16.99 | 1457.24 | 1607.9 | 1.46±0.16 | 1.38±0.23 | 2.27±0.17 |
| 127 | .tau.-Cadinol | 5937-11-1 | 17.04 | 1481.76 | 1639 | 2.38±0.25 | 2.41±0.2 | 0±0 |
| 128 | 1-Hexadecanol | 36653-82-4 | 17.07 | 1488.111 | 1879 | 1.32±0.16 | 1.06±0.06 | 1.51±0.09 |
| 129 | .tau.-Muurolol | 19912-62-0 | 17.23 | 1490.355 | 1640 | 0.54±0.07 | 0±0 | 1.81±0.18 |
| 130 | Adipic acid, butyl isobutyl ester | 1000324-09-3 | 17.24 | 1507.702 | - | 1.36±0.19 | 1.53±0.26 | 4±0.64 |
| 131 | 3-Ethyl-3-methylheptane | 17302-01-1 | 17.41 | 1519.709 | - | 1.11±0.12 | 0±0 | 0±0 |
| 132 | 1,1'-Biphenyl, 2,2',5,5'-tetramethyl- | 3075-84-1 | 17.67 | 1523.564 | 1663.6 | 1.36±0.18 | 1.46±0.2 | 3.09±0.45 |
| 133 | Heptadecane, 3-methyl- | 6418-44-6 | 17.80 | 1558.829 | 1771 | 0.95±0.09 | 0±0 | 0.52±0.04 |
| 134 | Neophytadiene | 504-96-1 | 17.84 | 1598.896 | 1840 | 0.65±0.11 | 0.67±0.07 | 1.75±0.05 |
| 135 | Caffeine | 58-08-2 | 17.86 | 1602.435 | 1842 | 6.41±1.28 | 0±0 | 31.7±12.11 |
| 136 | Phthalic acid, isobutyl 4-octyl ester | 1000314-84-7 | 18.01 | 1616.307 | - | 2.33±0.38 | 2.06±0.25 | 0±0 |
| 137 | 7,9-Di-tert-butyl-1-oxaspiro(4,5)deca-6,9-diene-2,8-dione | 82304-66-3 | 18.18 | 1645.22 | 1929 | 1.82±0.3 | 0±0 | 2.35±0.47 |
| 138 | Phthalic acid, 6-ethyl-3-octyl butyl ester | 1000315-17-4 | 18.35 | 1601.215 | - | 1.23±0.27 | 1.16±0.06 | 0.53±0.11 |
| 139 | Hexadecanoic acid, ethyl ester | 628-97-7 | 18.49 | 1659.326 | 1993 | 2.61±0.37 | 1.7±0.16 | 0.98±0.05 |
| 140 | Phytol | 150-86-7 | 18.66 | 1875.985 | 2122 | 1.59±0.33 | 1.78±0.3 | 7.14±1.75 |
| 141 | 9,12,15-Octadecatrienoic acid, ethyl ester, (Z,Z,Z)- | 1191-41-9 | 18.82 | 1974.87 | 2169 | 0.99±0.21 | 0.96±0.02 | 1.6±0.21 |
| 142 | Ethane, 1,1-diethoxy- | 105-57-7 | 18.82 | 774.1991 | 725 | 0±0 | 0.51±0.11 | 0±0 |
| 143 | 2,6-Dimethyl-1,3,5,7-octatetraene, E,E- | 460-01-5 | 18.84 | 1105.575 | 1134 | 0±0 | 0±0 | 3.1±0.23 |
| 144 | Isophthalaldehyde | 626-19-7 | 18.89 | 1142.249 | - | 0±0 | 0.83±0.06 | 1.32±0.11 |
| 145 | Naphthalene | 91-20-3 | 18.99 | 1148.094 | 1178 | 0±0 | 2.01±0.04 | 0±0 |
| 146 | L-.alpha.-Terpineol | 10482-56-1 | 19.32 | 1156.024 | 1187 | 0±0 | 1.31±0.03 | 3.72±0.25 |
| 147 | 1-Decen-3-one | 56606-79-2 | 19.50 | 1444.136 | - | 0±0 | 1.7±0.21 | 0±0 |

| | | | | | | | | |
|---|---|---|---|---|---|---|---|---|
| 148 | .alpha.-Cadinol | 481-34-5 | 19.73 | 1490.333 | 1650 | 0±0 | 0.83±0.09 | 0.79±0.09 |
| 149 | Sulfurous acid, 2-ethylhexyl hexyl ester | 1000309-20-2 | 19.81 | 1515.833 | - | 0±0 | 0.63±0.08 | 0±0 |
| 150 | 1,2-Benzenediol, o-(4-methoxybezoyl)-o'-(5-chlorovaleryl)- | 1000325-98-7 | 19.85 | 1066.903 | - | 0±0 | 0±0 | 0.81±0.27 |
| 151 | 2-Pentenal, (E)- | 1576-87-0 | 19.89 | 787.5302 | 754 | 0±0 | 0.8±0.27 | 0±0 |
| 152 | 1-Penten-3-one, 2-methyl- | 25044-01-3 | 20.04 | 797.1268 | - | 0±0 | 2.56±0.72 | 0±0 |
| 153 | 2-Propenenitrile | 107-13-1 | 20.16 | 815.9929 | - | 0±0 | 1.08±0.16 | 0±0 |
| 154 | Oxetane, 3-(1-methylethyl)- | 10317-17-6 | 20.29 | 838.8964 | - | 0±0 | 0.17±0.05 | 0±0 |
| 155 | Octatriene, 1,3-trans-5-trans- | 33580-04-0 | 20.43 | 881.3859 | - | 0±0 | 0.62±0.16 | 0±0 |
| 156 | Butanedioic acid, phenyl- | 635-51-8 | 20.50 | 890.642 | - | 0±0 | 2.29±0.23 | 0±0 |
| 157 | 4-Heptenal, (Z)- | 6728-31-0 | 21.00 | 899.8032 | 904 | 0±0 | 1.14±0.31 | 0±0 |
| 158 | 3-Octen-2-one | 1669-44-9 | 21.15 | 1026.203 | 1037 | 0±0 | 1.99±0.16 | 1.55±0.13 |
| 159 | 1-Decanol, 2-ethyl- | 21078-65-9 | 21.27 | 1042.565 | - | 0±0 | 5.31±1.08 | 0±0 |
| 160 | Ethanone, 1-(1H-pyrrol-2-yl)- | 1072-83-9 | 21.27 | 1045.791 | 1063.2 | 0±0 | 0.95±0.1 | 8.54±0.39 |
| 161 | Nonane, 4,5-dimethyl- | 17302-23-7 | 21.41 | 1045.696 | - | 0±0 | 1.77±1.75 | 0±0 |
| 162 | 2-n-Butyl furan | 4466-24-4 | 21.46 | 1087.4 | - | 0±0 | 2±0.25 | 0±0 |
| 163 | 2-Methylthiolane, S,S-dioxide | 1003-46-9 | 21.47 | 1104.461 | - | 0±0 | 0.72±0.15 | 0±0 |
| 164 | 1,3,8-p-Menthatriene | 18368-95-1 | 21.48 | 1105.219 | 1113 | 0±0 | 0.79±0.02 | 1.04±0.09 |
| 165 | Benzene, 1-isocyano-2-methyl- | 10468-64-1 | 21.48 | 1112.423 | - | 0±0 | 3.57±0.16 | 0±0 |
| 166 | Undecane, 4,4-dimethyl- | 17312-68-4 | 21.59 | 1117.415 | - | 0±0 | 0.77±0.03 | 0±0 |
| 167 | Butanoic acid, 3-hexenyl ester, (Z)- | 16491-36-4 | 21.91 | 1153.118 | 1186 | 0±0 | 1.53±0.09 | 0±0 |
| 168 | (S)-(+)-6-Methyl-1-octanol | 110453-78-6 | 21.91 | 1156.656 | - | 0±0 | 0.46±0.08 | 0±0 |
| 169 | 2-Nonanone, 3-(hydroxymethyl)- | 67801-33-6 | 22.11 | 1158.015 | 1093 | 0±0 | 0.52±0.02 | 0±0 |
| 170 | 2-Decenal, (E)- | 3913-81-3 | 22.22 | 1212.929 | 1265 | 0±0 | 1.53±0.32 | 1.69±0.49 |
| 171 | Dodecane, 2,6,10-trimethyl- | 3891-98-3 | 22.34 | 1233.909 | 1376 | 0±0 | 0.91±1.16 | 0±0 |
| 172 | 1-Oxaspiro[4.5]dec-6-ene, 2,6,10,10-tetramethyl- | 36431-72-8 | 22.43 | 1255.427 | - | 0±0 | 1.25±0.02 | 0±0 |
| 173 | Undecane, 3,7-dimethyl- | 17301-29-0 | 22.48 | 1277.324 | 1221 | 0±0 | 1.76±0.68 | 0±0 |
| 174 | Decane, 3,6-dimethyl- | 17312-53-7 | 22.70 | 1282.759 | - | 0±0 | 0.91±0.23 | 0±0 |
| 175 | 1, 1, 5-Trimethyl-1, 2-dihydronaphthalene | 1000357-25-8 | 22.70 | 1283.107 | - | 0±0 | 0.9±0.01 | 1.91±0.11 |
| 176 | Tetradecane, 2-methyl- | 1560-95-8 | 22.80 | 1295.146 | 1461 | 0±0 | 5.41±0.98 | 0±0 |
| 177 | Tetradecane, 3-methyl- | 18435-22-8 | 23.07 | 1320.14 | 1468 | 0±0 | 0.67±0.13 | 0±0 |
| 178 | Carbonic acid, decyl undecyl ester | 1000383-16-0 | 23.28 | 1381.948 | - | 0±0 | 1.11±0.47 | 0±0 |

| | | | | | | | | |
|---|---|---|---|---|---|---|---|---|
| 179 | Hexadecane, 2,6,11,15-tetramethyl- | 504-44-9 | 23.43 | 1414.716 | - | 0±0 | 2±2.05 | 2.43±0.12 |
| 180 | Glutaric acid, di(isobutyl) ester | 1000358-25-0 | 23.89 | 1439.712 | - | 0±0 | 4.93±0.56 | 0±0 |
| 181 | 1,7-Dimethyl-4-(1-methylethyl)cyclodecane | 645-10-3 | 24.03 | 1448.275 | - | 0±0 | 0.39±0.1 | 0±0 |
| 182 | Naphthalene, 1,6-dimethyl-4-(1-methylethyl)- | 483-78-3 | 24.24 | 1503.131 | 1674 | 0±0 | 0.55±0.02 | 1.1±0.07 |
| 183 | Pentadecane, 2,6,10,14-tetramethyl- | 1921-70-6 | 24.53 | 1519.688 | 1707 | 0±0 | 1.34±0.05 | 2.24±0.22 |
| 184 | 1,2-Benzenediol, o-(4-methoxybenzoyl)-o'-(2,2,3,3,4,4,4-heptafluorobutyryl)- | 1000325-97-9 | 24.54 | 1414.716 | - | 0±0 | 0.61±0.03 | 0±0 |
| 185 | 6-Octen-1-ol, 3,7-dimethyl-, propanoate | 141-14-0 | 24.61 | 1623.302 | 1446 | 0±0 | 0.44±0.03 | 0±0 |
| 186 | Hexadecanoic acid, methyl ester | 112-39-0 | 24.75 | 1647.697 | 1920 | 0±0 | 0.83±0.02 | 4.5±0.39 |
| 187 | 1-Hepten-3-one | 2918-13-0 | 24.90 | 971.8068 | - | 0±0 | 0±0 | 5.85±1.17 |
| 188 | Decyl octyl ether | 1000406-38-3 | 24.98 | 1042.955 | - | 0±0 | 0±0 | 3.84±0.28 |
| 189 | 1,5,7-Octatrien-3-ol, 3,7-dimethyl- | 29957-43-5 | 24.99 | 1085.354 | 1107 | 0±0 | 0±0 | 69.78±3.35 |
| 190 | Benzyl nitrile | 140-29-4 | 25.17 | 1112.795 | 1140 | 0±0 | 0±0 | 9.94±0.22 |
| 191 | Phenol, 4-amino-2,5-dimethyl- | 3096-71-7 | 25.32 | 1149.957 | - | 0±0 | 0±0 | 4.82±0.24 |
| 192 | Benzaldehyde, 4-methyl- | 104-87-0 | 25.48 | 1179.391 | 1079 | 0±0 | 0±0 | 5±0.58 |
| 193 | Naphthalene, 1,2,3,4-tetrahydro-1,1,6-trimethyl- | 475-03-6 | 25.50 | 1286.118 | 1258 | 0±0 | 0±0 | 0.41±0.03 |
| 194 | 1-Isopropyl-4,7-dimethyl-1,2,3,4,5,6-hexahydronaphthalene | 16729-00-3 | 25.59 | 1371.247 | 1481.3 | 0±0 | 0±0 | 1.46±0.14 |
| 195 | Di-epi-1,10-cubenol | 73365-77-2 | 25.80 | 1474.231 | 1619 | 0±0 | 0±0 | 1.76±0.18 |
| 196 | Phthalic acid, hept-4-yl isobutyl ester | 1000356-78-3 | 25.94 | 1616.589 | - | 0±0 | 0±0 | 0.45±0.07 |
| 197 | Linoleic acid ethyl ester | 544-35-4 | 26.15 | 1963.9 | 2155 | 0±0 | 0±0 | 0.17±0.02 |
| 198 | Cyclopentene, 3-methyl- | 1120-62-3 | 26.15 | 739.3248 | - | 0±0 | 0±0 | 0.72±0.09 |
| 199 | Acetic anhydride | 108-24-7 | 26.28 | 816.8185 | - | 0±0 | 0±0 | 2.82±0.12 |
| 200 | 1H-Imidazole-4-methanol | 822-55-9 | 26.29 | 866.2909 | - | 0±0 | 0±0 | 0.6±0.42 |
| 201 | 1,3-trans,5-cis-Octatriene | 40087-61-4 | 26.38 | 881.6126 | 880 | 0±0 | 0±0 | 0.64±0.06 |
| 202 | Pyrazine, 2,5-dimethyl- | 123-32-0 | 26.71 | 909.2551 | 913 | 0±0 | 0±0 | 1.53±0.07 |
| 203 | Ethanone, 1-(2-furanyl)- | 1192-62-7 | 26.73 | 910.2027 | 914 | 0±0 | 0±0 | 2.95±0.08 |
| 204 | Pyrazine, ethyl- | 13925-00-3 | 26.84 | 913.3619 | 916 | 0±0 | 0±0 | 1.39±0.16 |
| 205 | 2-Furancarboxaldehyde, 5-methyl- | 620-02-0 | 26.89 | 957.8428 | 966 | 0±0 | 0±0 | 2.67±0.15 |
| 206 | Benzonitrile | 100-47-0 | 27.04 | 976.0085 | 994 | 0±0 | 0±0 | 2.14±0.15 |
| 207 | Phenol | 108-95-2 | 27.04 | 977.4303 | 981 | 0±0 | 0±0 | 1.5±0.04 |
| 208 | Pyrazine, 2-ethyl-6-methyl- | 13925-03-6 | 27.06 | 991.1283 | 1001 | 0±0 | 0±0 | 4.38±0.1 |
| 209 | Bicyclo[2.2.1]hept-2-ene, 1,7,7-trimethyl- | 464-17-5 | 27.20 | 1005.012 | - | 0±0 | 0±0 | 1.07±0.15 |
| 210 | .gamma.-Terpinene | 99-85-4 | 27.35 | 1043.207 | 1062 | 0±0 | 0±0 | 1.67±0.06 |
| 211 | Ethyl 4-(ethyloxy)-2-oxobut-3-enoate | 1000305-38-2 | 27.35 | 1045.387 | - | 0±0 | 0±0 | 0.83±0.06 |
| 212 | Octane, 2-methyl- | 3221-61-2 | 27.36 | 1047.631 | - | 0±0 | 0±0 | 1.45±0.21 |
| 213 | Cyclobutanone, 2-methyl- | 1517-15-3 | 27.43 | 1055.244 | - | 0±0 | 0±0 | 7.29±0.14 |
| 214 | Pyrazine, 3-ethyl-2,5-dimethyl- | 13360-65-1 | 27.52 | 1061.827 | 1078 | 0±0 | 0±0 | 3.53±0.12 |

| | | | | | | | | |
|---|---|---|---|---|---|---|---|---|
| 215 | Benzene, 4-ethenyl-1,2-dimethyl- | 27831-13-6 | 27.59 | 1070.315 | - | 0±0 | 0±0 | 1.99±0.09 |
| 216 | 1-(2,3-Dimethylphenyl)ethanone | 2142-71-4 | 27.66 | 1070.915 | - | 0±0 | 0±0 | 0.46±0.06 |
| 217 | Butane, 2-iodo-3-methyl- | 18295-27-7 | 28.27 | 1086.808 | - | 0±0 | 0±0 | 6.54±0.39 |
| 218 | 2,5-Pyrrolidinedione, 1-ethyl- | 2314-78-5 | 28.53 | 1110.156 | - | 0±0 | 0±0 | 0.71±0.05 |
| 219 | 2,4,6-Octatriene, 2,6-dimethyl- | 673-84-7 | 28.76 | 1115.243 | 1113 | 0±0 | 0±0 | 0.85±0.04 |
| 220 | 2,6,6-Trimethyl-2-cyclohexene-1,4-dione | 1125-21-9 | 28.84 | 1116.981 | 1142 | 0±0 | 0±0 | 0.37±0.02 |
| 221 | 5-Hydroxy-7-methoxy-2-methyl-3-phenyl-4-chromenone | 55927-39-4 | 28.84 | 1135.148 | - | 0±0 | 0±0 | 0.26±0.14 |
| 222 | 2-Chloroethyl benzoate | 939-55-9 | 29.29 | 1139.572 | - | 0±0 | 0±0 | 1.5±2.24 |
| 223 | Benzeneacetic acid, methyl ester | 101-41-7 | 29.46 | 1146.143 | 1179 | 0±0 | 0±0 | 0.81±0.04 |
| 224 | d-Proline, N-methoxycarbonyl-, heptyl ester | 1000320-79-1 | 29.60 | 1148.45 | - | 0±0 | 0±0 | 1.96±0.14 |
| 225 | Butanoic acid, 3-hexenyl ester, (E)- | 53398-84-8 | 29.74 | 1153.442 | 1185.9 | 0±0 | 0±0 | 1.38±0.11 |
| 226 | 1-Phenyl-2-butanone | 1007-32-5 | 29.76 | 1185.353 | - | 0±0 | 0±0 | 0.47±0.03 |
| 227 | Benzeneacetic acid | 103-82-2 | 29.88 | 1203.71 | 1254 | 0±0 | 0±0 | 0.63±0.18 |
| 228 | Citral | 5392-40-5 | 29.88 | 1208.766 | - | 0±0 | 0±0 | 67.68±2.83 |
| 229 | Benzeneacetaldehyde, .alpha.-ethylidene- | 4411-89-6 | 30.02 | 1222.29 | 1281 | 0±0 | 0±0 | 0.64±0.04 |
| 230 | Sulfurous acid, 2-ethylhexyl undecyl ester | 1000309-19-4 | 31.04 | 1238.468 | - | 0±0 | 0±0 | 2.69±0.38 |
| 231 | 4-Acetoxy-3-methoxystyrene | 46316-15-8 | 31.27 | 1253.477 | - | 0±0 | 0±0 | 0.64±0.07 |
| 232 | Decane, 3,8-dimethyl- | 17312-55-9 | 31.28 | 1277.219 | 1113 | 0±0 | 0±0 | 2.07±0.09 |
| 233 | Formic acid, decyl ester | 5451-52-5 | 31.42 | 1369.414 | - | 0±0 | 0±0 | 0.41±0.05 |
| 234 | 10-Methylnonadecane | 56862-62-5 | 32.69 | 1391.281 | - | 0±0 | 0±0 | 1.28±0.36 |
| 235 | Phenol, 2,5-bis(1,1-dimethylethyl)- | 5875-45-6 | 32.82 | 1396.369 | 1514 | 0±0 | 0±0 | 2.8±0.34 |
| 236 | .alpha.-Calacorene | 21391-99-1 | 32.83 | 1418.583 | 1522 | 0±0 | 0±0 | 4.55±0.38 |
| 237 | 1,6,10-Dodecatrien-3-ol, 3,7,11-trimethyl-, (E)- | 40716-66-3 | 33.31 | 1430.876 | 1551 | 0±0 | 0±0 | 1.59±0.18 |
| 238 | Glutaric acid, butyl isobutyl ester | 1000358-25-1 | 33.32 | 1440.142 | - | 0±0 | 0±0 | 9.21±1.05 |
| 239 | 1-Tetradecanol | 112-72-1 | 33.56 | 1448.667 | 1676 | 0±0 | 0±0 | 0.52±0.1 |
| 240 | Dodecanoic acid, ethyl ester | 106-33-2 | 34.34 | 1450.531 | 1579 | 0±0 | 0±0 | 0.78±0.07 |
| 241 | Decyl heptyl ether | 1000406-39-1 | 34.43 | 1511.898 | - | 0±0 | 0±0 | 1.46±0.26 |
| 242 | Heptadecane | 629-78-7 | 35.16 | 1516.195 | - | 0±0 | 0±0 | 0.97±0.11 |
| 243 | Cyclohexane, undecyl- | 54105-66-7 | 35.17 | 1552.283 | - | 0±0 | 0±0 | 1.22±0.12 |
| 244 | 3,5-di-tert-Butyl-4-hydroxybenzaldehyde | 1620-98-0 | 35.75 | 1559.204 | - | 0±0 | 0±0 | 0.22±0.02 |
| 245 | 9H-Fluorene, 9-methylene- | 4425-82-5 | 37.93 | 1562.964 | - | 0±0 | 0±0 | 0.69±0.03 |
| 246 | 2-Pentadecanone, 6,10,14-trimethyl- | 502-69-2 | 38.82 | 1602.622 | 1838 | 0±0 | 0±0 | 2.14±0.24 |
| 247 | Phthalic acid, butyl hept-4-yl ester | 1000356-78-4 | 38.93 | 1601.815 | - | 0±0 | 0±0 | 3.76±0.82 |

$RI^A$: the linear retention indices calculated from a series of n-alkanes (C7-C40).
$RI^{B:}$ retention indices referred to the literature value with same or equivalent chromatographic column shown on NIST Chemistry WebBook (http://webbook.nist.gov/chemistry/).

**Table S3**. List aroma characteristics and OAV values of 41 different volatile compounds in different second-drying temperature samples.

| Name | OT[A] | Aroma characteristics[B] | BHL70 | BHL90 | BHL110 |
|---|---|---|---|---|---|
| Toluene | 140 | Sweet | 0.022±0.006 | 0.01±0.003 | 0.017±0.001 |
| (E)-3-Hexen-1-ol | 110 | Mushroom-like odor | 0.072±0.012 | 0.061±0.007 | 0.026±0.003 |
| Ethylbenzene | 29 | Aromatic odor | 0.059±0.015 | 0±0 | 0±0 |
| 1-Hexanol | 0.7 | Green, cut grass | 10.139±1.893 | 2.366±0.259 | 4.125±0.4 |
| Styrene | 730 | aromatic odour | 0.005±0.002 | 0±0 | 0.003±0 |
| 2-Heptanone | 1 | pieplant, musty | 2.15±0.614 | 0±0 | 1.34±0.145 |
| (E)-4-Heptenal | 0.02 | green | 67.075±15.333 | 0±0 | 28.552±2.95 |
| Heptanal | 3 | Heavy, planty green odor | 5.942±1.245 | 3.977±0.621 | 0.705±0.053 |
| Benzaldehyde | 3 | almond-like smelle | 2.269±0.288 | 1.043±0.091 | 1.237±0.03 |
| cis-2-(2-Pentenyl)furan | 6 | Bean-like, fruity, green, earthy and vegetable-like smell | 0.151±0.037 | 0±0 | 0±0 |
| Octanal | 0.7 | Green, fatty, citruse | 5.878±1.491 | 3.955±0.54 | 0±0 |
| (E,E)-2,4-Heptadienal | 56 | faint sweet, faint pungent fruity | 0.13±0.028 | 0.114±0.023 | 0.02±0.001 |
| D-Limonene | 10 | citrus herbal terpene camphore | 0.335±0.055 | 0.178±0.04 | 0.711±0.076 |
| 3-Carene | 5 | Leafy | 0.583±0.116 | 0.66±0.055 | 0±0 |
| 1-Octanol | 0.022 | Green, citrus, fatty, coconut-like | 533.649±89.582 | 450.988±37.786 | 0±0 |
| Ethyl 2-(5-methyl-5-vinyltetrahydrofuran-2-yl)propan-2-yl carbonate | 320 | Roasted, sweet | 0.015±0.002 | 0.027±0.001 | 0.026±0.002 |
| Nonanal | 1 | candle-like, sweet orange-like, fatty and floral odord | 29.965±4.338 | 29.623±1.381 | 0±0 |
| Phenylethyl Alcohol | 564.23 | floral, sweet, rose | 0.027±0.003 | 0.017±0.002 | 0.047±0.002 |
| alpha-Terpineol | 300 | Citrus aromas, woody | 0.005±0.001 | 0±0 | 0±0 |
| 2,6,6-trimethyl-1,3-Cyclohexadiene-1-carboxaldehyde | 3 | Woody, spicy, medicinal, powdery, herbal | 0.707±0.156 | 0.519±0.003 | 0.881±0.058 |
| Decanal | 0.1 | Aldehyde-like, candle-like, fatty and citrus-like aroma | 18.955±2.782 | 27.893±2.264 | 16.438±0.336 |
| (Z)-3,7-dimethyl-2,6-Octadien-1-ol | 0.0002 | Citrus, lemon-like | 328487.048±39485.402 | 0±0 | 337756.081±14275.132 |
| 1-Decanol | 0.023 | Orange, Floral | 25.13±7.387 | 0±0 | 0±0 |
| Indole | 140 | animal-like | 0.006±0.001 | 0.086±0.01 | 0.039±0.002 |
| 2-methyl-Naphthalene | 10 | piquancy | 0.066±0.007 | 0.092±0 | 0.071±0.002 |
| 1-Dodecanol | 5 | Violet-like, oil | 0.222±0.023 | 0±0 | 0±0 |
| Nerolidol 2 | 0.25 | Floral, green, citrus, woody, waxy | 11.083±0.739 | 6.981±0.669 | 0±0 |
| (Z)-3-Hexen-1-ol, benzoate | 12.1 | green, fruity, florald | 0.045±0.004 | 0.049±0.004 | 0.074±0.007 |
| Cedrol | 0.5 | mild cedar wood-like aroma | 2.925±0.311 | 2.77±0.451 | 4.531±0.349 |
| tau-Cadinol | 0.44 | Tar, camphor, and greasy | 5.416±0.56 | 5.487±0.443 | 0±0 |
| Phytol | 640 | Floral, balsam, powdery, waxy | 0.002±0.001 | 0.003±0 | 0.011±0.003 |
| Naphthalene | 0.44 | Pungent, dry, tarry odore | 0±0 | 4.571±0.094 | 0±0 |
| 1-(1H-pyrrol-2-yl)-Ethanone | 170000 | nutty | 0±0 | 0±0 | 0±0 |
| 2,6,10,10-tetramethyl-1-Oxaspiro[4.5]dec-6-ene | 0.2 | Fruity, woody, slightly camphor-like | 0±0 | 6.251±0.111 | 0±0 |
| 3,7-dimethyl-1,5,7-Octatrien-3-ol | 110 | moldy | 0±0 | 0±0 | 0.634±0.03 |
| 2,5-dimethyl-Pyrazine | 1750 | Roasted | 0±0 | 0±0 | 0.001±0 |
| ethyl-Pyrazine | 4000 | Nutty coffee, cocoa-like | 0±0 | 0±0 | 0±0 |
| 5-methyl-2-Furancarboxaldehyde | 500 | Caramel, bready, coffee-like | 0±0 | 0±0 | 0.005±0 |
| 2-ethyl-6-methyl-Pyrazine | 40 | Roasted | 0±0 | 0±0 | 0.11±0.002 |
| 3-ethyl-2,5-dimethyl-Pyrazine | 8.6 | Roasted potato, cocoa-like, nutty | 0±0 | 0±0 | 0.41±0.014 |
| trans-beta-Ionone | 0.007 | Violet-like, floral, and raspberry-like | 9676.937±1134.502 | 9260.629±769.571 | 10176.878±591.999 |

OTs: Odor thresholds in water. The values were according to the reported references.
A: (Wang et al., 2020a; Wang et al., 2020b; Guo et al., 2021a; Guo et al., 2021b); Zhu et al., 2018; Zhu et al., 2021; Zhang et al., 2019)
B: http://www.thegoodscentscompany.com/search3.php?qOdor=20126-76-5&submit.x=9&submit.y=9

## 2.2 Supplementary Figures

**Figure. S1** Evolution of compounds from different sources, (A) Fatty derived volatiles, (B) Glycoside-derived volatiles, (C) Amino acid-derived volatiles, (D) Carotenoid-derived volatiles

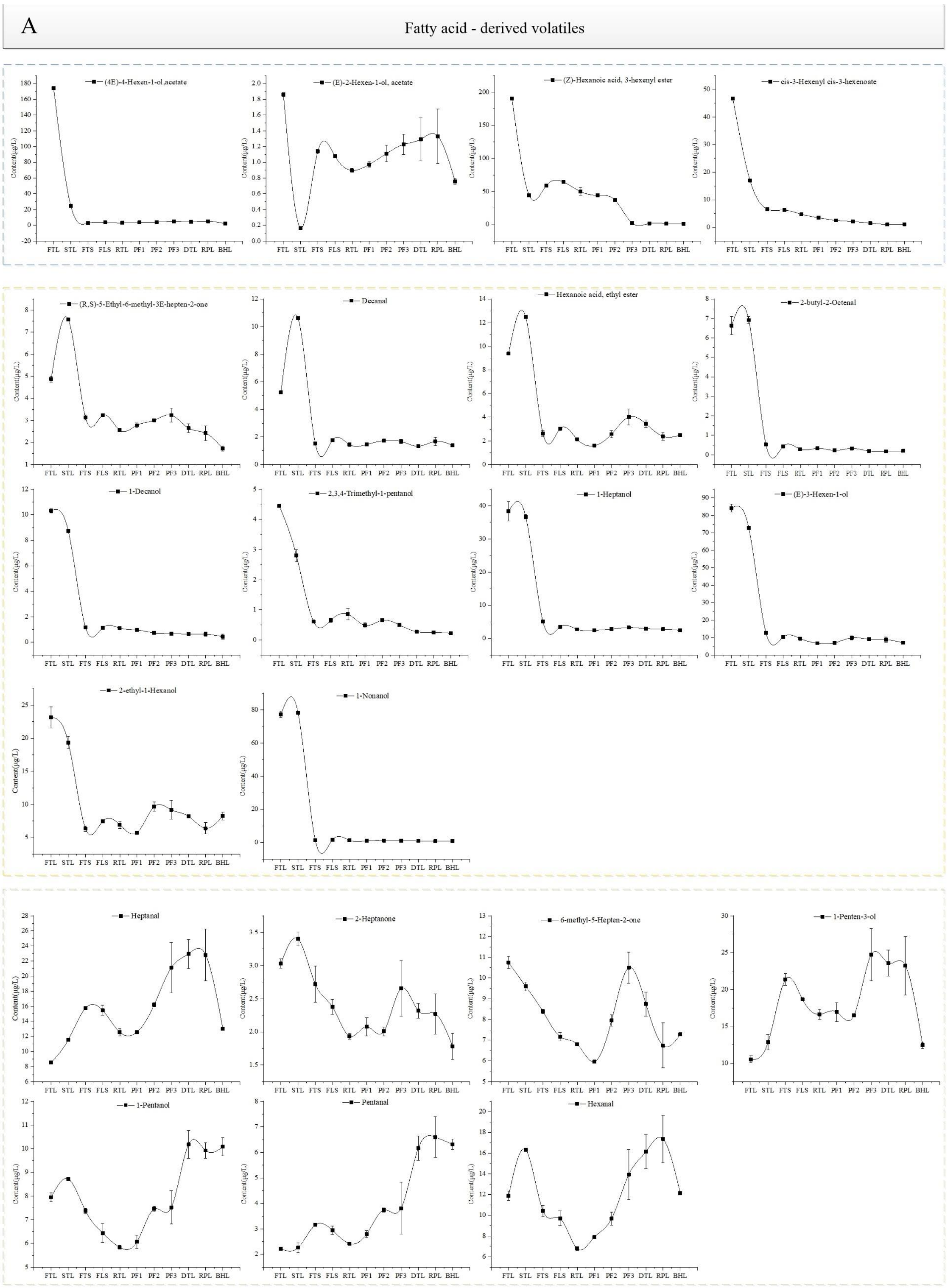

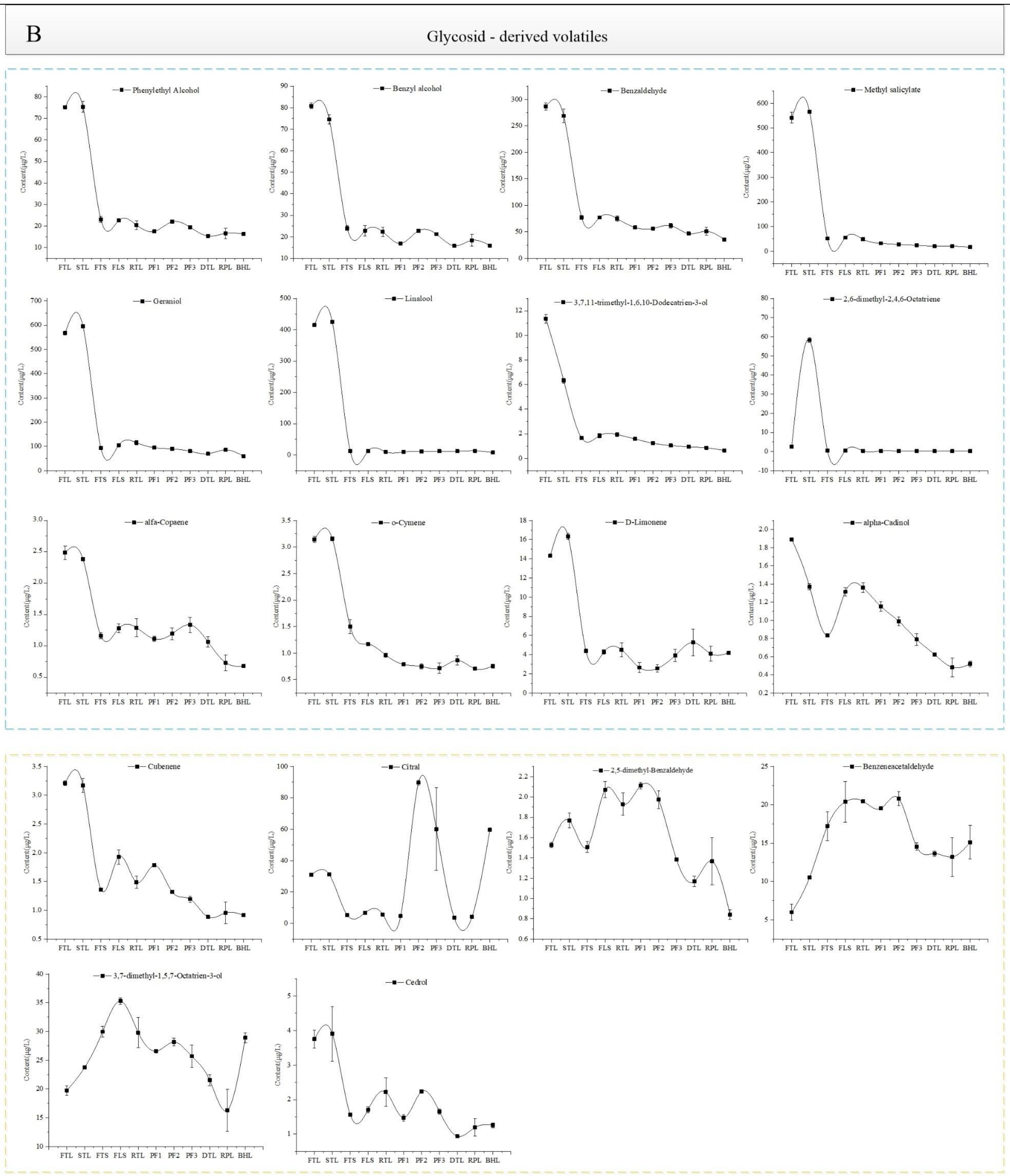
B
Glycosid - derived volatiles
Phenylethyl Alcohol
Benzyl alcohol
Benzaldehyde
Methyl salicylate
Geraniol
Linalool
3,7,11-trimethyl-1,6,10-Dodecatrien-3-ol
2,6-dimethyl-2,4,6-Octatriene
alfa-Copaene
o-Cymene
D-Limonene
alpha-Cadinol
Cubenene
Citral
2,5-dimethyl-Benzaldehyde
Benzeneacetaldehyde
3,7-dimethyl-1,5,7-Octatrien-3-ol
Cedrol
Content(μg/L)
FTL STL FTS FLS RTL PF1 PF2 PF3 DTL RPL BHL

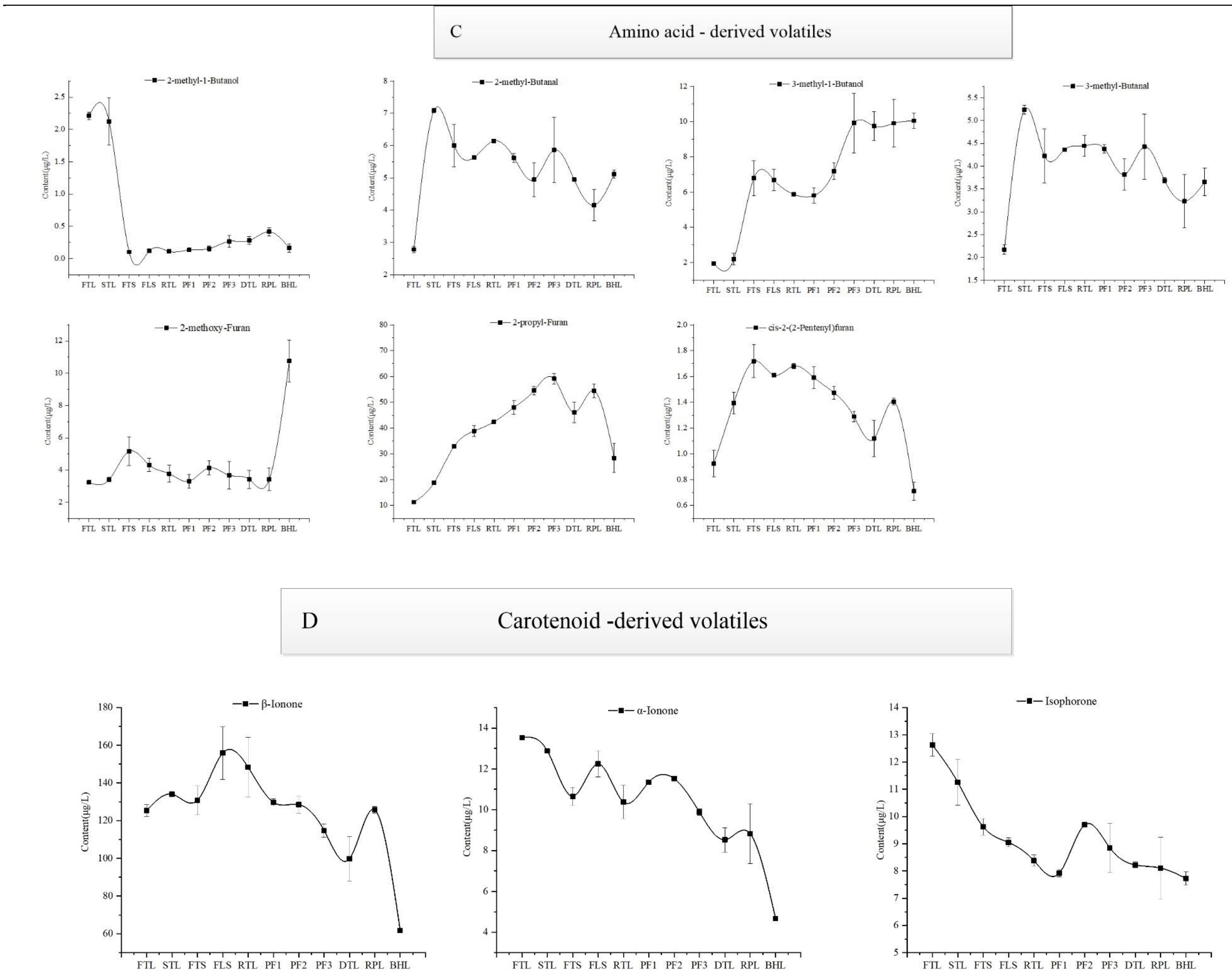


Figure. S2 Heat map of different volatile compounds screened out in different second-drying temperature

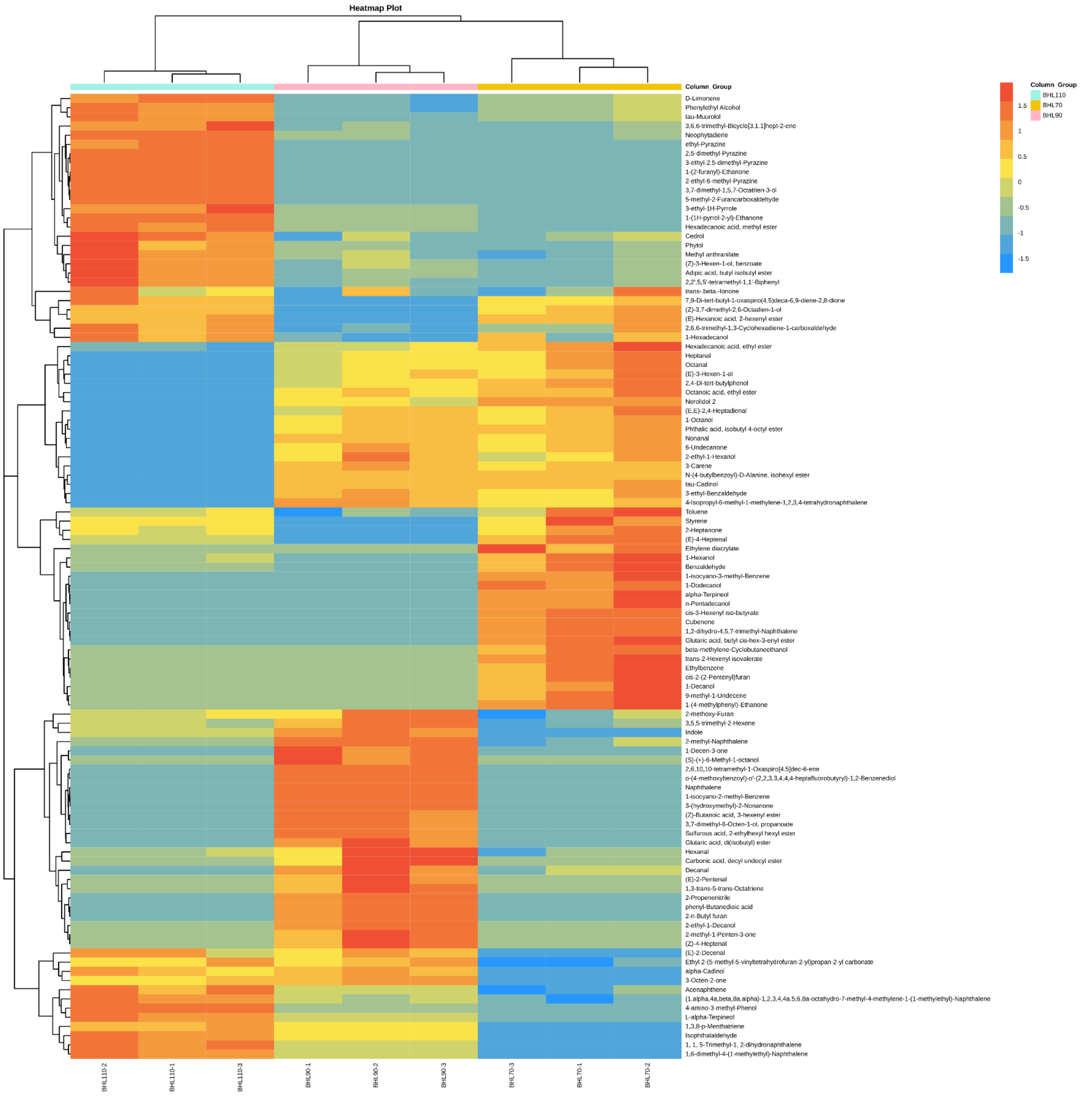

Heatmap Plot
Column_Group
D-Limonene
Phenylethyl Alcohol
tau-Muurolol
3,6,6-trimethyl-Bicyclo[3.1.1]hept-2-ene
Neophytadiene
ethyl-Pyrazine
2,5-dimethyl-Pyrazine
3-ethyl-2,5-dimethyl-Pyrazine
1-(2-furanyl)-Ethanone
2-ethyl-6-methyl-Pyrazine
3,7-dimethyl-1,5,7-Octatrien-3-ol
5-methyl-2-Furancarboxaldehyde
3-ethyl-1H-Pyrrole
1-(1H-pyrrol-2-yl)-Ethanone
Hexadecanoic acid, methyl ester
Cedrol
Phytol
Methyl anthranilate
(Z)-3-Hexen-1-ol, benzoate
Adipic acid, butyl isobutyl ester
2,2',5,5'-tetramethyl-1,1'-Biphenyl
trans-.beta.-Ionone
7,9-Di-tert-butyl-1-oxaspiro(4,5)deca-6,9-diene-2,8-dione
(Z)-3,7-dimethyl-2,6-Octadien-1-ol
(E)-Hexanoic acid, 2-hexenyl ester
2,6,6-trimethyl-1,3-Cyclohexadiene-1-carboxaldehyde
1-Hexadecanol
Hexadecanoic acid, ethyl ester
Heptanal
Octanal
(E)-3-Hexen-1-ol
2,4-Di-tert-butylphenol
Octanoic acid, ethyl ester
Nerolidol 2
(E,E)-2,4-Heptadienal
1-Octanol
Phthalic acid, isobutyl 4-octyl ester
Nonanal
6-Undecanone
2-ethyl-1-Hexanol
3-Carene
N-(4-butylbenzoyl)-D-Alanine, isohexyl ester
tau-Cadinol
3-ethyl-Benzaldehyde
4-Isopropyl-6-methyl-1-methylene-1,2,3,4-tetrahydronaphthalene
Toluene
Styrene
2-Heptanone
(E)-4-Heptenal
Ethylene diacrylate
1-Hexanol
Benzaldehyde
1-isocyano-3-methyl-Benzene
1-Dodecanol
alpha-Terpineol
n-Pentadecanol
cis-3-Hexenyl iso-butyrate
Cubenene
1,2-dihydro-4,5,7-trimethyl-Naphthalene
Glutaric acid, butyl cis-hex-3-enyl ester
beta-methylene-Cyclobutaneethanol
trans-2-Hexenyl isovalerate
Ethylbenzene
cis-2-(2-Pentenyl)furan
1-Decanol
9-methyl-1-Undecene
1-(4-methylphenyl)-Ethanone
2-methoxy-Furan
3,5,5-trimethyl-2-Hexene
Indole
2-methyl-Naphthalene
1-Decen-3-one
(S)-(+)-6-Methyl-1-octanol
2,6,10,10-tetramethyl-1-Oxaspiro[4.5]dec-6-ene
o-(4-methoxybenzoyl)-o'-(2,2,3,3,4,4,4-heptafluorobutyryl)-1,2-Benzenediol
Naphthalene
1-isocyano-2-methyl-Benzene
3-(hydroxymethyl)-2-Nonanone
(Z)-Butanoic acid, 3-hexenyl ester
3,7-dimethyl-6-Octen-1-ol, propanoate
Sulfurous acid, 2-ethylhexyl hexyl ester
Glutaric acid, di(isobutyl) ester
Hexanal
Carbonic acid, decyl undecyl ester
Decanal
(E)-2-Pentenal
1,3-trans-5-trans-Octatriene
2-Propenenitrile
phenyl-Butanedioic acid
2-n-Butyl furan
2-ethyl-1-Decanol
2-methyl-1-Penten-3-one
(Z)-4-Heptenal
(E)-2-Decenal
Ethyl 2-(5-methyl-5-vinyltetrahydrofuran-2-yl)propan-2-yl carbonate
alpha-Cadinol
3-Octen-2-one
Acenaphthene
(1,alpha,4a,beta,8a,alpha)-1,2,3,4,4a,5,6,8a-octahydro-7-methyl-4-methylene-1-(1-methylethyl)-Naphthalene
4-amino-3-methyl-Phenol
L-alpha-Terpineol
1,3,8-p-Menthatriene
Isophthalaldehyde
1, 1, 5-Trimethyl-1, 2-dihydronaphthalene
1,6-dimethyl-4-(1-methylethyl)-Naphthalene
BHL110-2
BHL110-1
BHL110-3
BHL90-1
BHL90-2
BHL90-3
BHL70-3
BHL70-1
BHL70-2
1.5
1
0.5
0
-0.5
-1
-1.5
Column_Group
BHL110
BHL70
BHL90
